# Single-cell RNA sequencing data analysis of the inner ear in gentamicin-treated mice via intraperitoneal injection

**DOI:** 10.1515/med-2025-1242

**Published:** 2025-11-21

**Authors:** Xiaolin Bao, Yuan Wang, Wei Liu, Huiling Tang, Yufen Guo

**Affiliations:** Department of Otorhinolaryngology Head and Neck Surgery, Tianjin TEDA Hospital, Tianjin, 300457, China; Department of Otorhinolaryngology Head and Neck Surgery, The Second Hospital of Lanzhou University, Lanzhou, No. 82 Cuiyingmen, ChengGuan District, Lanzhou, Gansu, 730030, China; Department of Otorhinolaryngology Head and Neck Surgery, The Second Hospital of Lanzhou University, Lanzhou, Gansu, 730030, China

**Keywords:** gentamicins, inner ear, single-cell RNA sequencing, ototoxicity, cochlear hair cells

## Abstract

**Objective:**

This study aims to delineate the mechanisms through which intraperitoneal injection of gentamicin (GEN) influences the inner ear cells of mice by employing single-cell RNA sequencing (scRNA-seq) technology.

**Methods:**

Eight-week-old Kunming mice were randomly assigned to three groups: a normal control group, a GEN group, and a GEN + dexamethasone (DEX) group. The mice received continuous intraperitoneal injections of the corresponding drugs for 10 days. Auditory brainstem response (ABR) was assessed to evaluate hearing threshold shifts, and cochlear tissues were harvested for scRNA-seq. The Seurat analysis workflow was employed for data quality control, dimensionality reduction clustering, and differential gene expression analysis.

**Results:**

ABR results demonstrated a significant elevation in hearing thresholds in the GEN group, whereas thresholds in the DEX group showed improvement but remained elevated compared to the NOR group (*P* < 0.05). Single-cell sequencing revealed notable alterations in the populations of outer hair cells, supporting cells, and immune cells in the GEN group. Analysis of differentially expressed genes identified significant downregulation of cell-specific genes Gbp6, Ppfia4 in hair cells of the GEN group, alongside upregulation of inflammation-related genes Nnat, Gh, indicating that hair cell damage and enhanced immune responses may be pivotal mechanisms underlying GEN-induced ototoxicity.

**Conclusion:**

Utilizing scRNA-seq technology, this study uncovers substantial transcriptional changes induced by GEN in cochlear hair cells, supporting cells, and immune cells in mice, highlighting the role of inflammation and oxidative stress, TNF signaling pathways in its ototoxicity. DEX partially ameliorates hair cell damage.

## 1 Introduction

The clinical application of ototoxic drugs may result in irreversible damage to the inner ear’s structure and function, manifesting as auditory and vestibular symptoms including hearing loss, tinnitus, and vertigo, which significantly impair patients’ quality of life [1]. Presently, the diagnosis of drug-induced hearing loss primarily depends on medical history, clinical presentations, and auditory function assessments; however, the absence of specific biomarkers poses substantial limitations on early detection and intervention [2,3].

Aminoglycosides, loop diuretics, antitumor agents (e.g., cisplatin), and salicylates are among the most common ototoxic drugs used clinically. Among them, aminoglycosides such as gentamicin (GEN) can induce cochlear hair cell damage, resulting in permanent hearing loss [4]. The pathogenic mechanisms are multifaceted, involving genetic predisposition, gene mutations, mitochondrial dysfunction, oxidative stress, and inflammatory responses [5–7]. Nonetheless, current research predominantly focuses on the whole tissue level, with limited analysis of the inner ear’s diverse cell types at a refined level. Specifically, the cell-specific transcriptomic changes induced by ototoxic drugs at single-cell resolution remain poorly understood, representing a considerable research gap [8,9].

GEN was chosen for this study due to its extensive clinical use and high ototoxicity risk among aminoglycosides. Widely prescribed worldwide for its broad-spectrum efficacy, stability, and low cost, GEN is especially prevalent in developing countries [10]. However, its clinical utility is hindered by a 10–25% incidence of irreversible hearing loss, even at therapeutic doses. GEN accumulates in cochlear tissues, particularly the stria vascularis (SV) and hair cells, where it disrupts mitochondrial function and induces oxidative stress. Its ototoxic effects are further amplified in pediatric patients, individuals with mitochondrial mutations, and those receiving concurrent ototoxic agents like loop diuretics [10–12]. Given its high-risk profile, GEN serves as an ideal model for studying aminoglycoside-induced hearing loss.

The advent of single-cell RNA sequencing (scRNA-seq) technology has provided a novel approach to unravel the cytological and molecular mechanisms underlying drug-induced hearing loss [13,14]. By extracting mouse inner ear tissue for scRNA-seq, coupled with cell clustering analysis, cell type identification, differential gene expression (DEG) analysis, and functional enrichment analysis, the susceptible cell types affected by GEN can be precisely identified, and their gene expression change profiles, along with key damage pathways, can be uncovered [15,16]. And it allows for the identification of rare or previously uncharacterized cell types, such as specific subpopulations of hair cells, supporting cells, or immune cells, which are often masked in bulk RNA-seq [17]. However, scRNA-seq is technically challenging when applied to small, calcified tissues like the cochlea due to difficulties in achieving high-quality single-cell suspensions. Transcript dropout, low capture efficiency, and batch effects can also affect interpretation. Despite these challenges, scRNA-seq remains a powerful tool for elucidating the complex cellular landscape of the inner ear, and is especially relevant in the context of ototoxicity and regeneration research, where cellular heterogeneity plays a key role [18]. This paves the way for screening potential biomarkers, offering a scientific foundation for the early diagnosis and monitoring of drug-induced hearing loss [19,20].

This study addresses three key questions using scRNA-seq: (1) How does GEN alter gene expression in specific cochlear cell types (e.g., outer hair cells [OHC], supporting cells, immune cells)? (2) Which molecular pathways (e.g., inflammation, oxidative stress, apoptosis) are primarily involved? (3) Can dexamethasone (DEX) mitigate these effects by modulating gene expression or inflammatory responses? These findings aim to clarify GEN’s ototoxic mechanisms and guide strategies for clinical risk reduction.

## 2 Materials and methods

### 2.1 Experimental animals and grouping

The study was approved by the Ethics Committee of Tianjin TEDA Hospital and conducted in strict accordance with ethical guidelines for animal experiments. Forty-five 8-week-old Kunming mice (KM, Mus musculus), regardless of sex, were housed in a specific-pathogen-free barrier conditions with controlled temperature (22 ± 2°C) and humidity (50 ± 5%), under a 12:12 h light cycle (light/dark), and provided with free access to food and water.

GEN is associated with high rates of ototoxicity. To counter the irreversible hearing loss, DEX was included in this study due to its well-documented protective mechanisms. DEX exerts anti-inflammatory effects by suppressing pro-inflammatory cytokines (e.g., tumor necrosis factor-alpha [TNF-α], IL-1β) and modulating nuclear factor-kappa B signaling, a key pathway involved in GEN-induced inflammation. In GEN-exposed cochlear explants, DEX has been shown to reduce TNF-α-mediated apoptosis by downregulating caspase-3 activation and preserving OHC viability [21,22]. Additionally, DEX exhibits anti-apoptotic properties by inhibiting the mitochondrial apoptotic pathway, specifically suppressing Bax overexpression, preventing cytochrome c release, and reducing caspase-3 activation. These effects collectively prevent GEN-triggered hair cell death, supporting DEX as a potential otoprotective agent [22]. Given these dual protective mechanisms, DEX serves as an ideal intervention for assessing its ability to mitigate GEN-induced ototoxicity.

The mice were randomly divided into three groups (*n* = 15):

(1) GEN group: Intraperitoneal injection of GEN at 200 mg/kg daily for 10 consecutive days, the dose and duration were selected based on previous studies [23,24].
(2) GEN + DEX group: Intraperitoneal injection of GEN at 200 mg/kg plus DEX sodium phosphate at 5 mg/kg daily for 10 consecutive days.
(3) Normal control group (NOR group): Intraperitoneal injection of 0.2 mL sterilized water daily for 10 consecutive days.

During the experiment, the general behavior of the mice (e.g., activity level, gait, and posture) was monitored daily, and body weight changes were recorded. Neurological reflexes were evaluated using the air righting reflex and tail suspension test, while auditory brainstem response (ABR) thresholds and rotarod performance were assessed before and after the experiment to evaluate vestibular function.

At the conclusion of the experiment, ten mice per group (totaling 30 mice, 30 cochleae in total) were used for scRNA-seq. Each sequencing sample was obtained from an individual mouse cochlea to ensure independent biological replicates.

The cochleae of the remaining five mice per group (totaling 15 mice) were used for histological and imaging analyses, including pathological sectioning, scanning electron microscopy (SEM), and histopathological examination of the cochlea (HE staining). Each experiment was performed on separate biological samples to ensure independent observations.

### 2.2 ABR testing in mice

ABR measurements were performed using the Tucker-Davis Technologies (TDT) auditory system (TDT, Alachua, FL, USA) and Biosig software. All experiments were carried out in a sound-attenuating chamber, and mice were anesthetized with 1% pentobarbital sodium (0.04 mL/g, intraperitoneal injection) prior to testing.

ABR signals were recorded using needle electrodes:

Recording electrode: placed subcutaneously at the midpoint of the line connecting the anterior edges of the two ears.

Reference electrode: placed subcutaneously behind the test ear.

Ground electrode: placed subcutaneously behind the contralateral ear.

The loudspeaker was placed approximately 1 cm from the entrance of the external auditory canal. The stimulus was a click sound (primary frequency: 12 kHz) with a band-pass filter ranging from 30 to 3,000 Hz, and signals were averaged over 512 repetitions with a scan time of 10 ms.

The steps of ABR testing are as follows:

(1) The initial sound intensity was set at 90 dB sound pressure level (SPL), decreasing in 10 dB increments until no repeatable ABR waveform was detected.
(2) The intensity was then increased in 5 dB increments until a repeatable ABR waveform reappeared.
(3) The lowest intensity at which wave II could be identified as the auditory threshold.
(4) The latencies of waves I and III, as well as the inter-peak intervals, were measured at 90 dB SPL.

### 2.3 Extraction of mouse cochlea

At the end of the experiments, mice were euthanized by carbon dioxide inhalation. Both ears were fixed, and the skull was excised using surgical scissors, following these steps to extract the cochlea:

(1) The skin was cut along the midline sagittal suture to expose the skull.
(2) The surrounding both ear canals were cut, and the occipital foramen was dissected open to separate the skull into left and right halves.
(3) Brain tissue on one side was removed, and the temporal bone was rapidly identified via the superior semicircular canal or uvula cerebelli, and then dissected out with micro-tweezers.
(4) Protocol for cell isolation, the cochlear tissues were transferred into a digestion enzyme solution containing 0.1–0.3% collagenase IV (w/v) and 0.05% trypsin (w/v) dissolved in Hanks’ Balanced Salt Solution, followed by incubation at 37°C for 15–30 min. Gentle pipetting was performed every 5 min to facilitate tissue dissociation. To terminate digestion, the reaction was quenched by adding culture medium supplemented with 10% fetal bovine serum. The cell suspension was filtered through a 40 μm cell strainer to remove undigested tissue debris. The filtrate was centrifuged at 300 × *g* for 5 min at 4°C, and the supernatant was discarded. The cell pellet was washed twice with phosphate-buffered saline. These details ensure reproducibility and clarify our workflow.
(5) For single-cell sequencing samples, the temporal bone was maintained on ice throughout the procedure. Fascia, nerves, and muscle tissues were removed, and the bone was immediately transferred to a 4°C storage solution for prompt scRNA-seq.

For pathological examination, the cochlea was fixed in 4% paraformaldehyde according to the following steps:

(1) The cochlea was exposed under a dissecting microscope, and surrounding tissue was cleared.
(2) The round window and oval window were opened to avoid damage to the basilar membrane.
(3) 4% paraformaldehyde was slowly injected through an apical opening until it exited from the round window.
(4) The cochlea was fixed in the refrigerator at 4°C for subsequent SEM analysis.

### 2.4 scRNA-seq

scRNA-seq in this study was performed with the assistance of Shanghai GeneChem Co., Ltd, utilizing the BD Rhapsody microplate sequencing technology. The experimental procedures included the following steps:

(1) Library preparation was carried out using the BD Rhapsody single-cell whole transcriptome amplification technology.
(2) cDNA sequencing was performed using Illumina 150 bp paired-end (PE150) sequencing.
(3) Raw data were processed using the BD Rhapsody Analysis Pipeline to obtain single-cell transcriptomic sequencing data.
(4) scRNA-seq was conducted using cochlear tissues from ten mice per group, ensuring independent biological replicates. Each scRNA-seq experiment was performed on a separate cochlea rather than pooled samples to maintain independent observations.
(5) Cell-type-specific marker genes were identified using the Seurat package by applying the FindAllMarkers function. This function utilizes the Wilcoxon rank-sum test to detect DEGs between each cell cluster and all other clusters, selecting genes with a minimum log-fold change threshold and statistical significance (adjusted *P*-value <0.05). Only genes exhibiting a cluster-specific expression pattern and meeting the predefined selection criteria were retained as marker genes.

### 2.5 Data statistics and analysis

Data analysis was performed using R software (R 4.2.0) with the Seurat package for single-cell clustering analysis, cell type identification, and DEGs analysis.

The statistical analysis procedures included the following steps:

(1) Cell clustering analysis involved t-distributed stochastic neighbour embedding (t-SNE) or uniform manifold approximation and projection (UMAP) dimensionality reduction methods for visualization.
(2) DEGs analysis was conducted using the Wilcoxon rank-sum test to identify DEGs (*P* < 0.05).
(3) Gene ontology (GO)/kyoto encyclopedia of genes and genomes (KEGG) enrichment analysis was performed using the ClusterProfiler package to analyze significantly enriched biological pathways.
(4) ABR data analysis was conducted with SPSS 26.0 software. Data are expressed as mean ± standard deviation ( \[\bar{x}]\] ± *s*), and group comparisons were performed using one-way analysis of variance, where *P* < 0.05 indicated a statistically significant difference.

**Ethical approval:** The study was approved by the Ethics Committee of Tianjin TEDA Hospital and conducted in strict accordance with ethical guidelines for animal experiments.

## 3 Results

### 3.1 ABR testing results in mice

ABR testing was employed to evaluate the auditory threshold changes in mice. A minimal Wave II threshold of ≤25 dB SPL was defined as the criterion for normal hearing, with thresholds >25 dB SPL considered indicative of hearing impairment.

(1) NOR group: All auditory thresholds were below 25 dB SPL, with a mean threshold of 22 dB SPL, indicating normal hearing.
(2) GEN + DEX group: After 10 days, five mice (33.3%) retained normal hearing with an average auditory threshold of 30.5 dB SPL, which was significantly elevated compared to the NOR group (*P* < 0.05).
(3) GEN group: Only two mice (13.3%) retained normal hearing, with an average auditory threshold of 38.5 dB SPL, demonstrating significant hearing loss (*P* < 0.01, compared to the NOR group). (Supplementary ABR-figure).

Statistical analysis revealed that the auditory thresholds in the GEN group were significantly higher than those in the NOR and DEX groups (*P* < 0.01), suggesting marked ototoxic damage induced by GEN, while DEX could partially mitigate hearing loss (*P* < 0.05).

The results of body weight changes across experimental groups and neurological reflex test results (air righting reflex and tail suspension test) are shown in Supplementary 1. The representative images and analysis of histopathological examination of the cochlea (HE staining), and representative images and analysis of SEM findings are shown in Supplementary HE stain-figure and SEM-figure.

### 3.2 scRNA-seq results

In the present study, scRNA-seq was performed on inner ear tissues from the three groups of mice using the BD Rhapsody platform. A total of 7,206 cells from the NOR group, 6,201 cells from the GEN group, and 6,990 cells from the DEX group were detected. Following data quality control (QC) and batch effect removal, the samples were included in the analysis.

#### 3.2.1 Data pre-processing – QC and data normalization

Setting of data QC criteria:

QC was applied to the raw sequencing data to exclude low-quality cells (with insufficient gene counts), potentially damaged cells (with high mitochondrial gene content), and outlier cells (with abnormal gene counts). The filtering criteria were as follows, based on previous studies [6,16,25,26]:

- 500 < Number of genes (nFeature_RNA) <6,000
- Number of transcripts (nCount_RNA) <25,000
- Mitochondrial gene percentage (mito%) <20%
- Ribosomal gene proportion >1% (indicating cell activity)
- Hemoglobin gene proportion <1% (excluding the impact of blood contamination)

**Figure 1:**
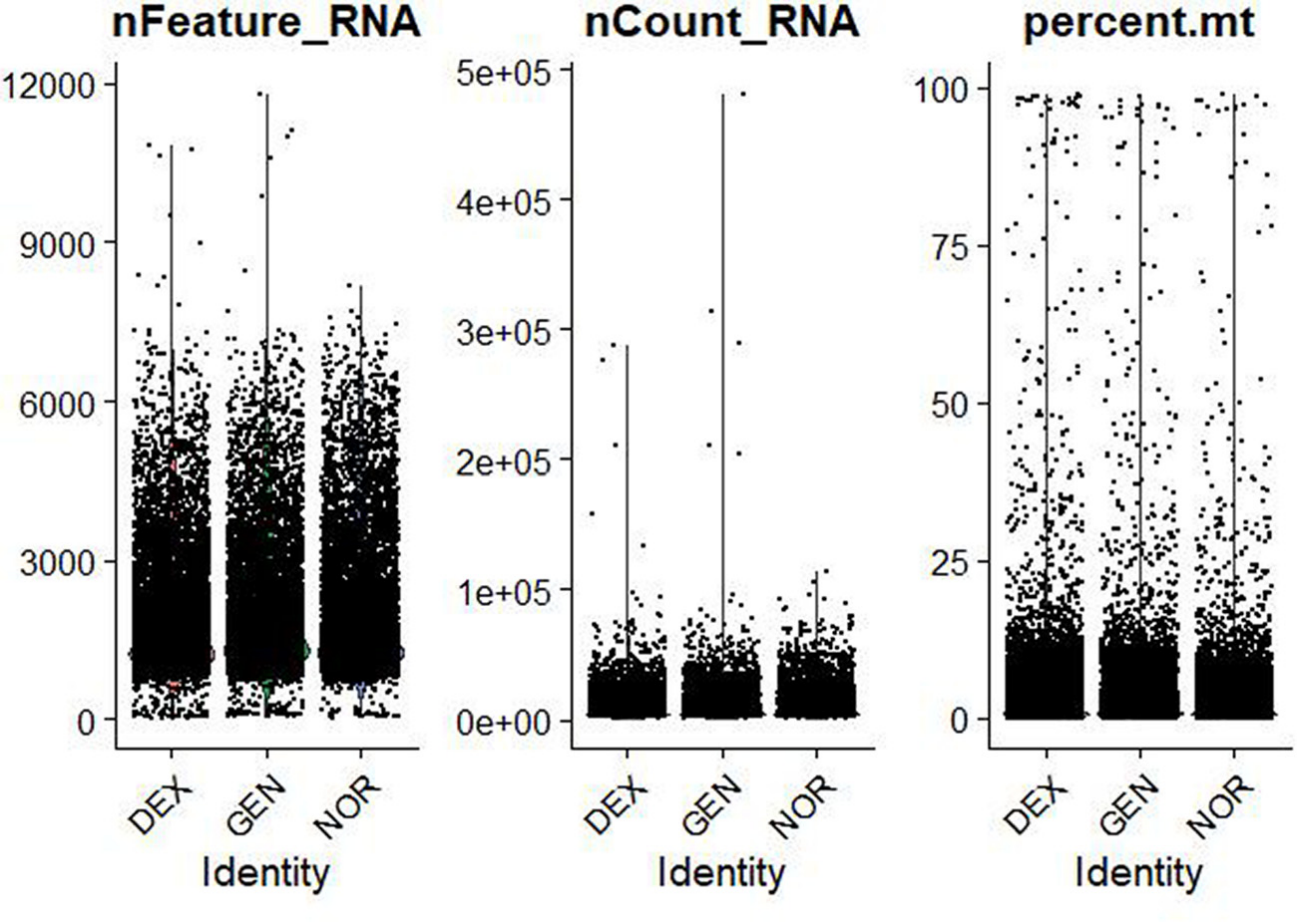
Violin plots of raw single‐cell QC metrics across DEX, GEN, and NOR groups prior to filtering. Violin plots of raw QC metrics (nFeature_RNA, nCount_RNA, percent.mt, percent_ribo, percent_Hb) across DEX, GEN, and NOR groups before filtering. These distributions illustrate the presence of empty droplets (<500 genes, <1,000 UMIs), doublets/high-content cells (>8,000 genes, >50,000 UMIs), high mitochondrial content (up to 60%), and blood contamination (percent_Hb > 1%).

**Figure 2:**
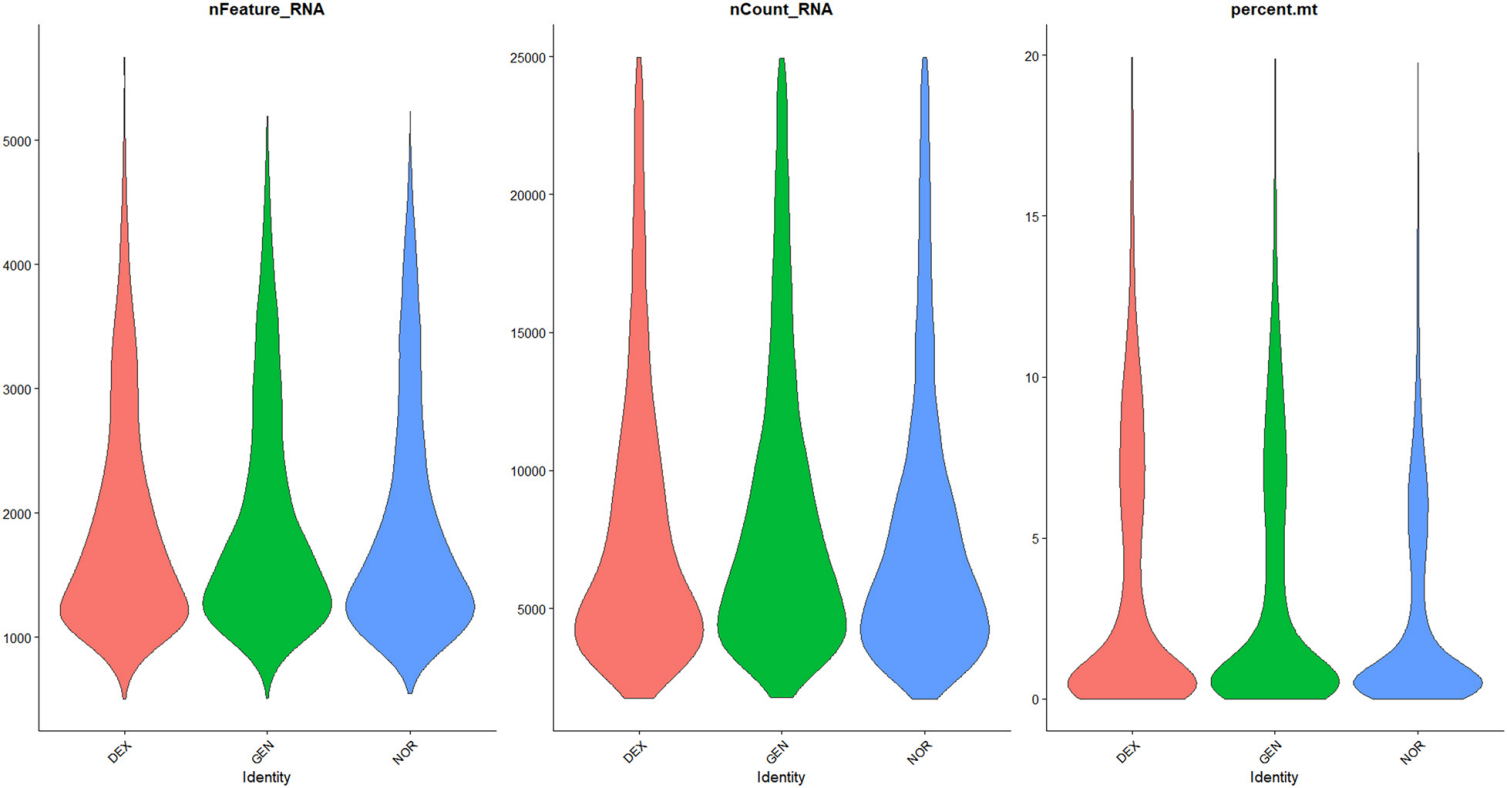
Violin plots of filtered single‐cell QC metrics across DEX, GEN, and NOR groups after applying QC thresholds. Violin plots of QC metrics after applying cutoffs (500 < nFeature_RNA < 6,000; nCount_RNA < 25,000; percent.mt < 20%; percent_ribo > 1%; percent_Hb < 1%). The post-filter distributions collapse tightly within these thresholds, confirming removal of low-quality, apoptotic, and blood-contaminated cells while retaining transcriptionally active single cells for downstream analysis.

**Figure 3:**
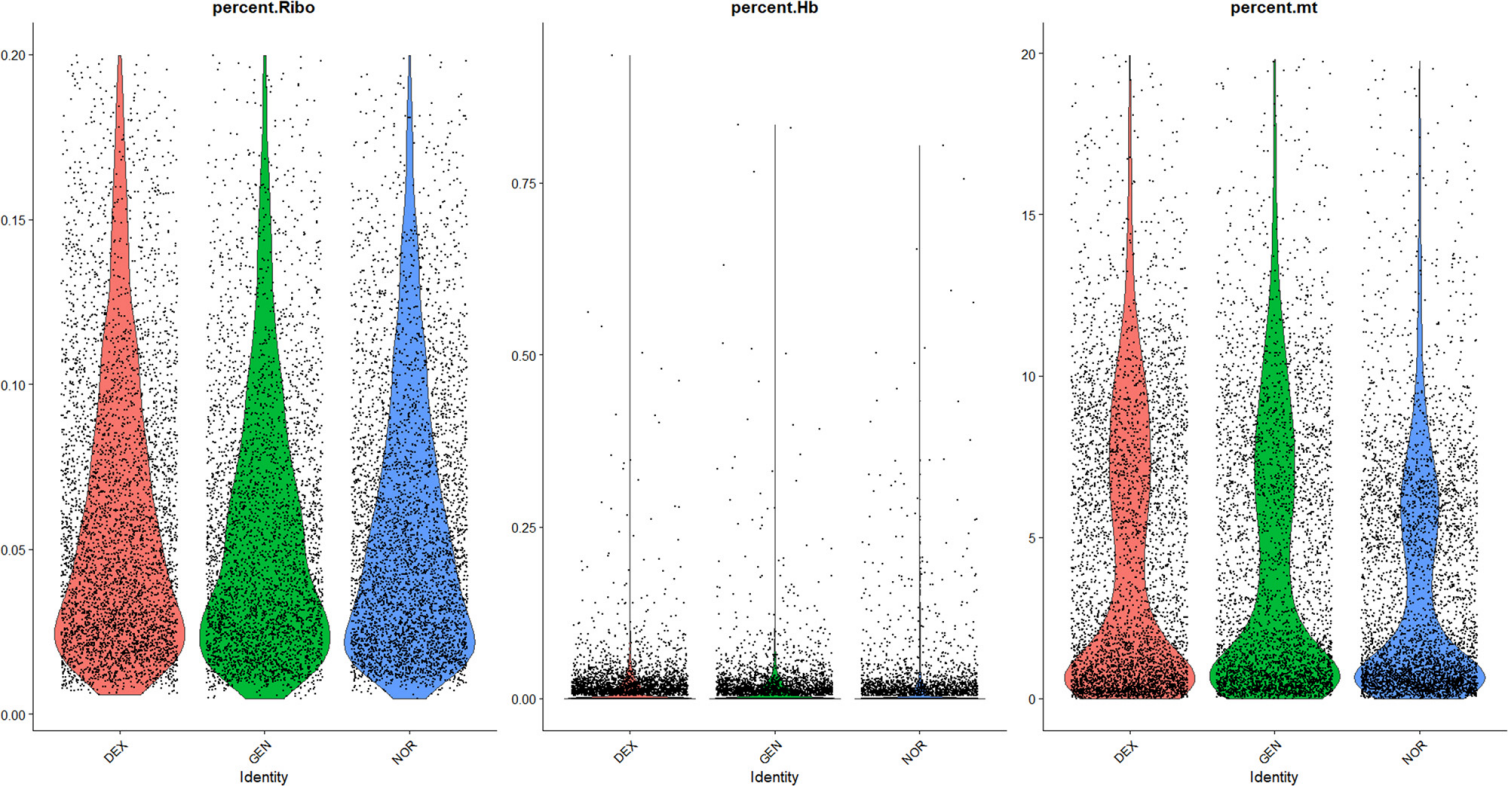
Comparative analysis of ribosomal genes, erythrocyte genes, and mitochondrial genes.

The data distribution before and after filtering is shown in Figures 1–3, highlighting a significant enhancement in data quality post-filtering. PostQC, hemoglobin gene transcripts constituted <0.5% of total reads, confirming minimal blood contamination.

#### 3.2.2 Cell cycle analysis

**Figure 4:**
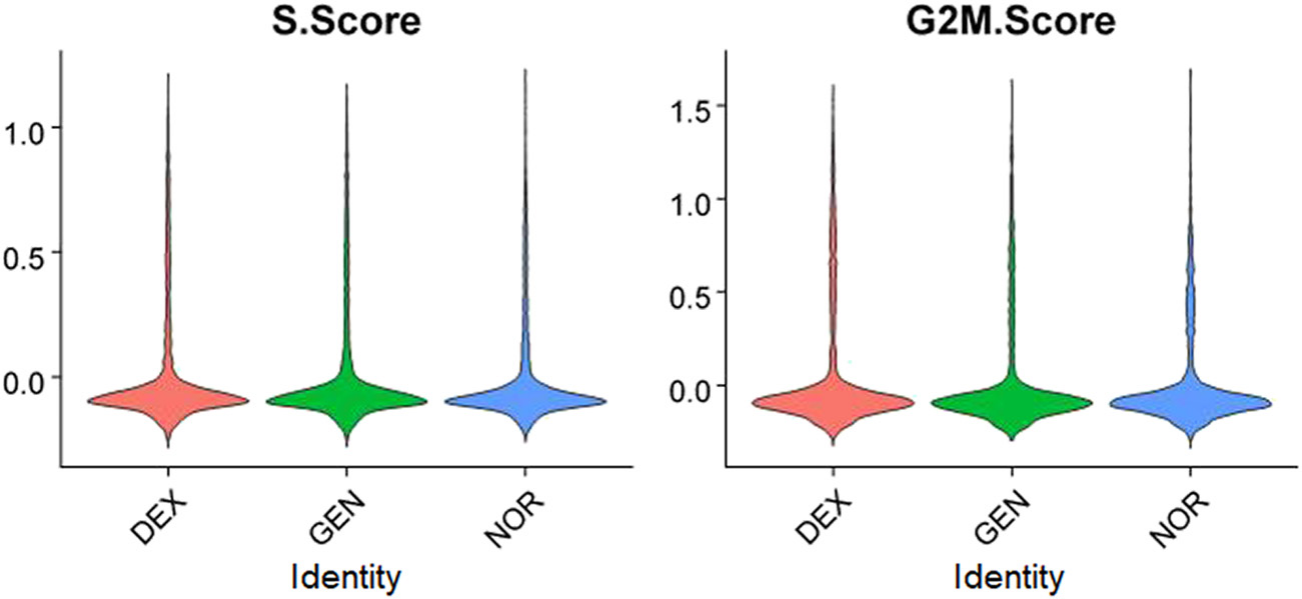
Cell cycle distribution of inner ear cells in different groups.

Assessment of cell cycle states:

Given the potential influence of cell cycle status on gene expression differences, cell cycle analysis was performed on data from all three groups. Results indicated that the majority of inner ear cells in mice resided primarily in the S and G2M phases, with cell cycle scores centralized around zero, suggesting no significant intergroup differences in cell cycle status, thereby not impeding subsequent analyses (Figure 4).

#### 3.2.3 Cell clustering analysis

##### 3.2.3.1 Feature gene selection and dimensionality reduction clustering

**Figure 5:**
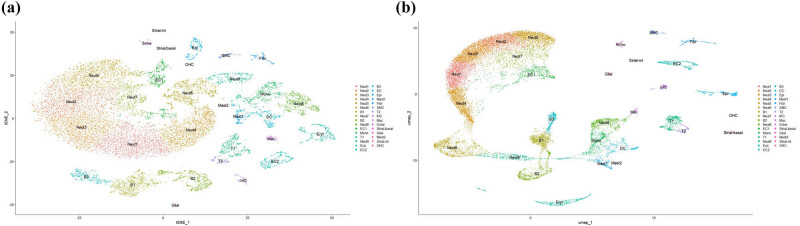
UMAP and t-SNE visualizations of Harmony‐corrected single‐cell data demonstrating batch integration across DEX, GEN, and NOR samples. (a) UMAP visualization of Harmony-corrected single-cell data across DEX, GEN, and NOR samples. (b) t-SNE visualization of the same integrated dataset, highlighting batch alignment.

High-variable genes were selected for clustering analysis based on Seurat’s highly variable gene selection. Principal component analysis (PCA) identified the top 20 principal components, followed by t-SNE and UMAP dimensionality reduction for visualization of clustering (Figure 5).

##### 3.2.3.2 Cell population classification

Clustering analysis identified a total of 31 cell populations, encompassing hair cells (OHC, inner hair cells), supporting cells (pillar cells, Deiters’ cells), immune cells (B cells, T cells, dendritic cells, macrophages), strial vascular cells (basal cells, marginal cells, intermediate cells), glial cells, Schwann cells, epithelial cells, among others.

#### 3.2.4 Cell type annotation – DEG analysis

##### 3.2.4.1 Cell type identification

The characteristic genes for each cell cluster were queried using a single-cell transcriptome database, and combined with known cell-specific marker genes to determine cell types (Table 1).

**Table 1:** Cell types and proportions and Chi-square statistic in the three groups

Cell type	NOR (%)	DEX (%)	GEN (%)	Chi-squared	*P*_val	*P* < 0.01
B cell	8.00	21.84	12.34	368.19	2.2 × 10^−16^	Yes
Dendritic cell	2.89	2.70	2.55	4.3898	0.1114	No
Endothelial cell	3.03	2.19	3.98	12.382	0.002048	Yes
Epithelial cell	2.78	1.82	1.51	25.799	0.0000025	Yes
Erythrocyte	0.13	0.18	0.33	2.8889	0.2359	No
Fibroblast	1.94	1.19	1.66	8.7246	0.01275	No
Glial cell	0.18	0.24	0.56	7.9512	0.01877	No
Hair cell	0.15	1.40	0.77	48.851	2.466 × 10^−11^	Yes
Macrophagecyte	0.75	0.94	1.02	1.0769	0.5836	No
Mastocyte	2.50	1.56	2.58	9.8526	0.007253	Yes
Monocyte	0.86	0.55	0.97	3.5882	0.1663	No
Neutrophil	68.98	60.69	63.65	71.474	3.018 × 10^−16^	Yes
Schwann cell	0.53	0.59	0.66	0.18182	0.9131	No
Smooth muscle cell	1.46	0.61	0.97	17.636	0.000148	Yes
Strial vascular	0.53	0.83	0.64	4.2069	0.122	No
T cell	5.28	2.66	5.80	43.107	4.359 × 10^−16^	Yes
Total	100.00	100.00	100.00	60.406	7.637 × 10^−14^	Yes

##### 3.2.4.2 Cell type-specific gene expression

**Figure 6:**
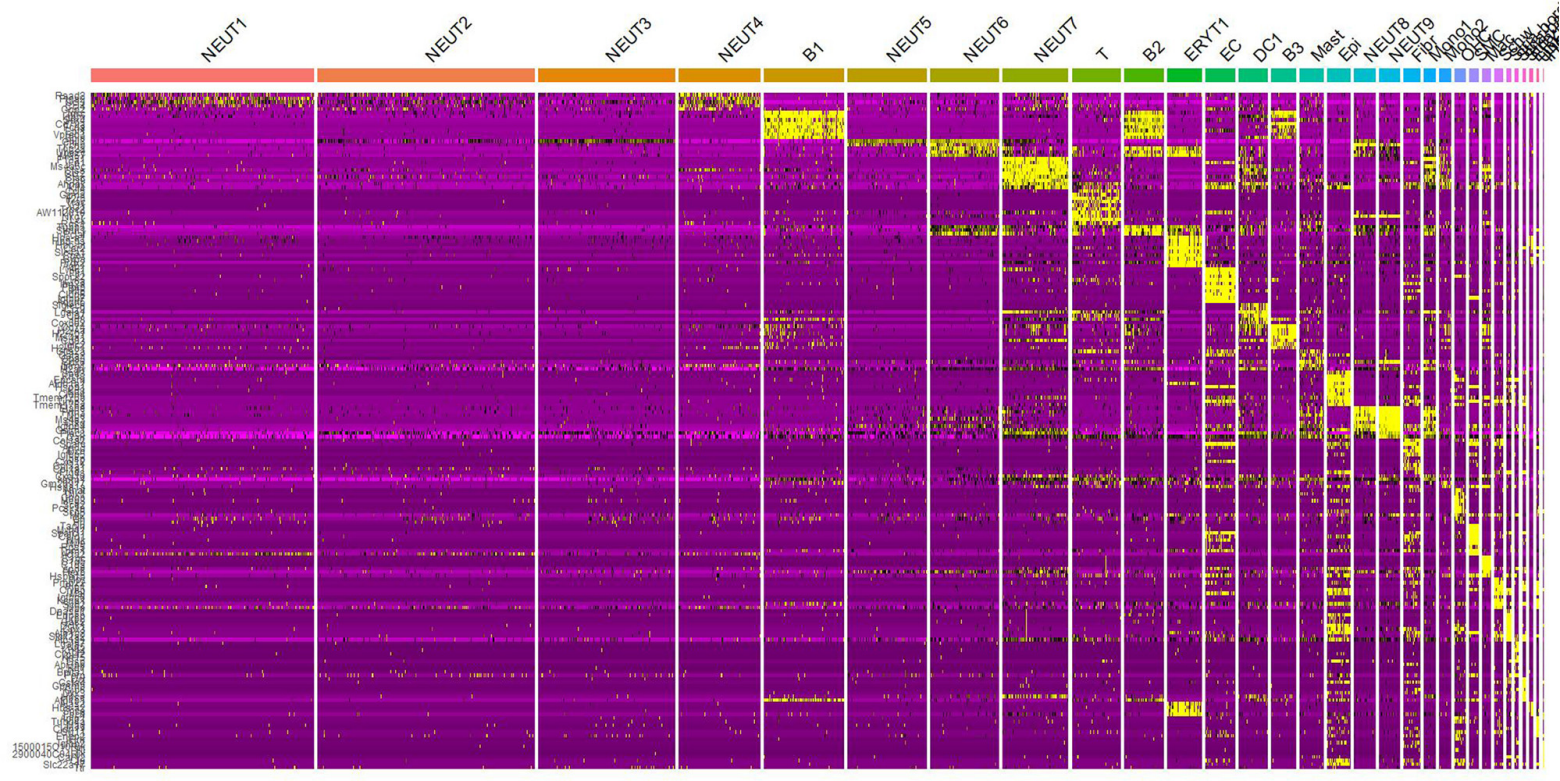
Heatmap of cell type-specific gene expression.

A heatmap of cell type-specific genes was generated (Figure 6) to further validate the accuracy of cell annotation. For example:

- OHC: highly express “Nnat”, “Dlk1”.
- B cells: highly express “Fcrla”, “Vpreb3”.
- T cells: highly express “Ccl5”, “Gzma”.

##### 3.2.4.3 DEG analysis

**Figure 7:**
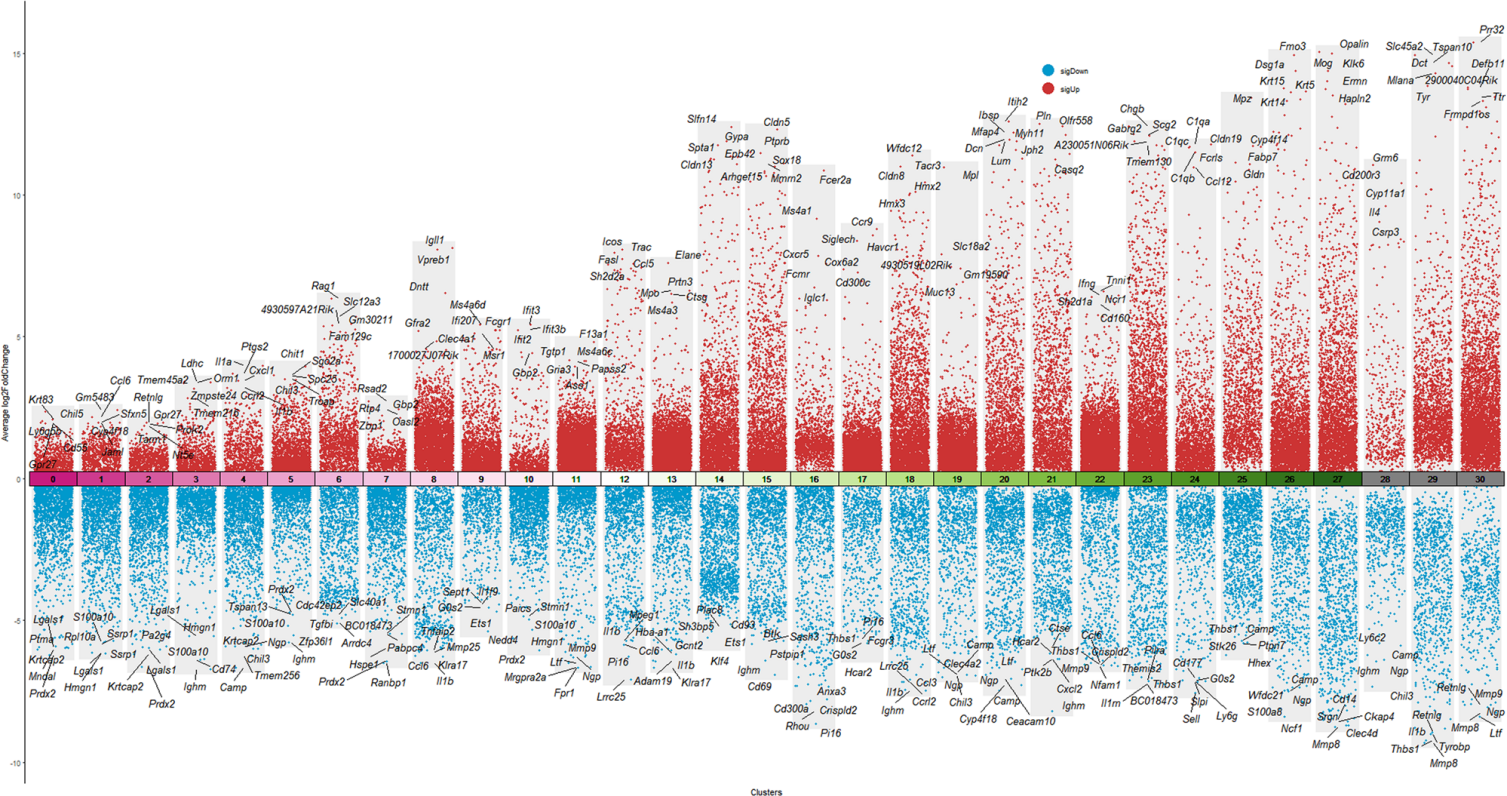
Volcano plot of DEGs between GEN and NOR groups.

The Wilcoxon rank-sum test was used to identify DEGs (*P* < 0.05), and a volcano plot depicting the top ten most upregulated genes was generated (Figure 7). Results indicate that, compared to the NOR group, expression of hair cell-specific genes (such as “Gbp6” and “Igfbpl1”) in the GEN group was significantly downregulated, whereas inflammation-related genes (such as “Gh” and “Nnat”) were markedly upregulated. The gene distribution visualizations of “Gbp6”, “Igfbpl1”, “Gh”, and “Nnat” are showed in Supplementary 3.

##### 3.2.4.4 Batch effect removal and data integration

① Batch effect removal

**Figure 8:**
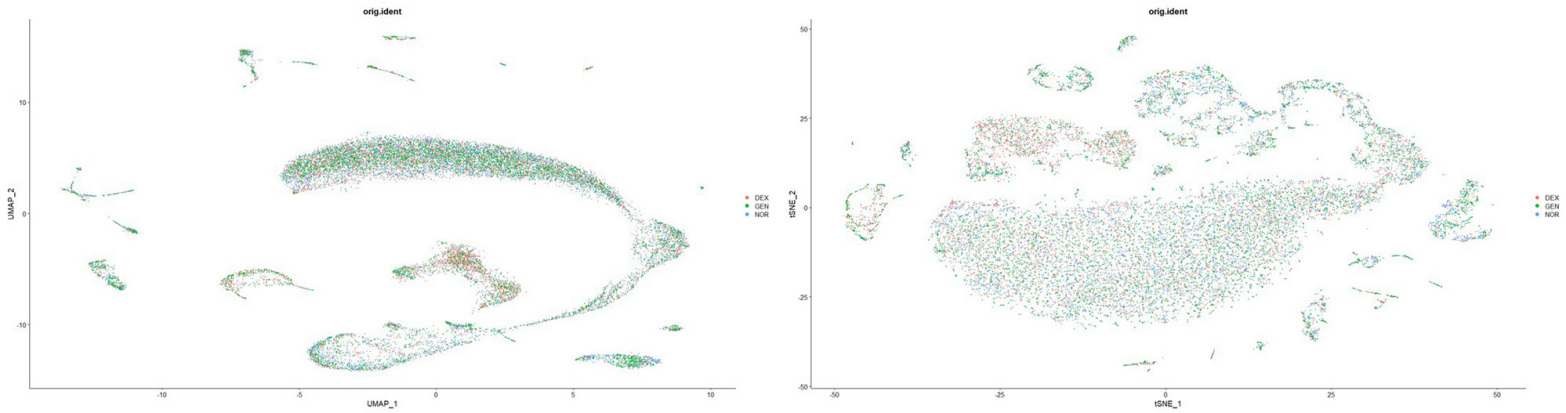
Batch effect removal and data integration results.

To mitigate technical variability arising from differences in sampling time, operators, and reagent lots, we applied the Harmony algorithm to our merged single‐cell dataset for batch‐effect correction. Following integration, we re-computed PCA and performed UMAP on the corrected embeddings. As shown in Figure 8, Harmony successfully collapsed the three original acquisition batches (DEX, GEN, NOR) into a unified transcriptional landscape: cells from all batches now co‐localize within shared UMAP clusters, and previously batch‐segregated subpopulations merge seamlessly while preserving well‐defined hair cell, supporting cell, and immune cell clusters. This result confirms that biological variation, rather than technical artifacts, drives the observed cellular heterogeneity and validates the use of the integrated dataset for downstream differential expression and trajectory analyses.

② Cell composition analysis

The proportion of each cell type is detailed in Table 1. Comparisons among the three groups revealed:

- A significant decrease in the proportion of hair cells in the GEN group (*P* < 0.01), while increases were observed in B cells and Schwann cells (*P* < 0.05), suggesting an enhanced inflammatory response.
- In the DEX group, the proportion of hair cells increased relative to the GEN group (*P* < 0.05), indicating a protective effect of DEX on hair cells.

#### 3.2.5 Distribution characteristics of mitochondrial-related genes

##### 3.2.5.1 Mitochondrial gene analysis

**Figure 9:**
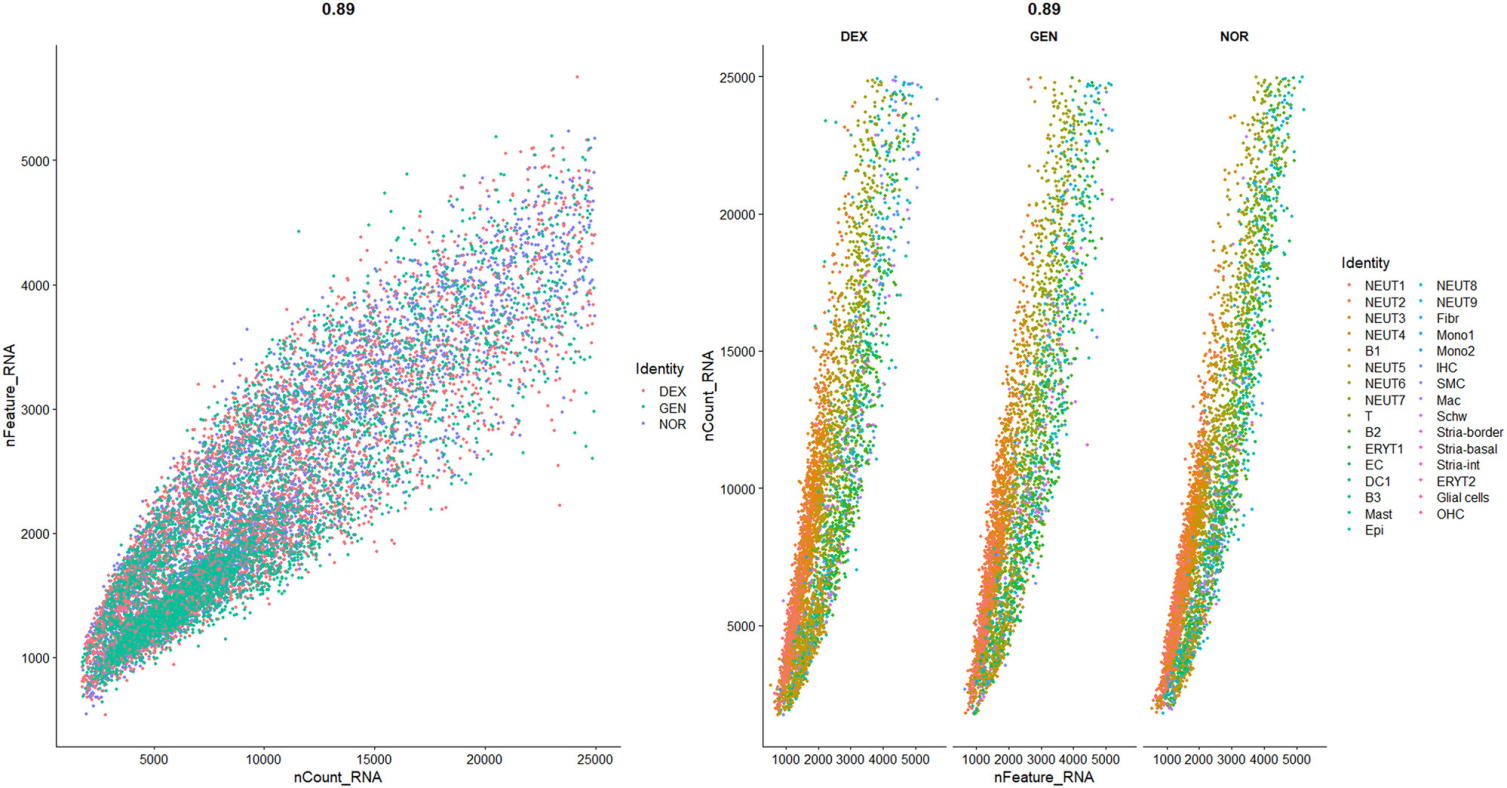
Expression of mitochondrial-related genes in different groups.

Based on data from the mouse mitochondrial gene database, a total of 1,140 mitochondrial genes were identified. Genes related to the oxidative phosphorylation (OXPHOS) pathway (ND1, ND2, CYTB) were significantly downregulated in the GEN group (*P* < 0.01), suggesting mitochondrial dysfunction as a critical mechanism of GEN-induced ototoxicity (Figure 9).

#### 3.2.6 Functional enrichment analysis

GO enrichment analysis of the DEGs revealed that genes upregulated in the GEN group were primarily enriched in pathways related to “inflammatory response”, “apoptosis”, and “oxidative stress” (*P* < 0.01, Figure 9).

**Figure 10:**
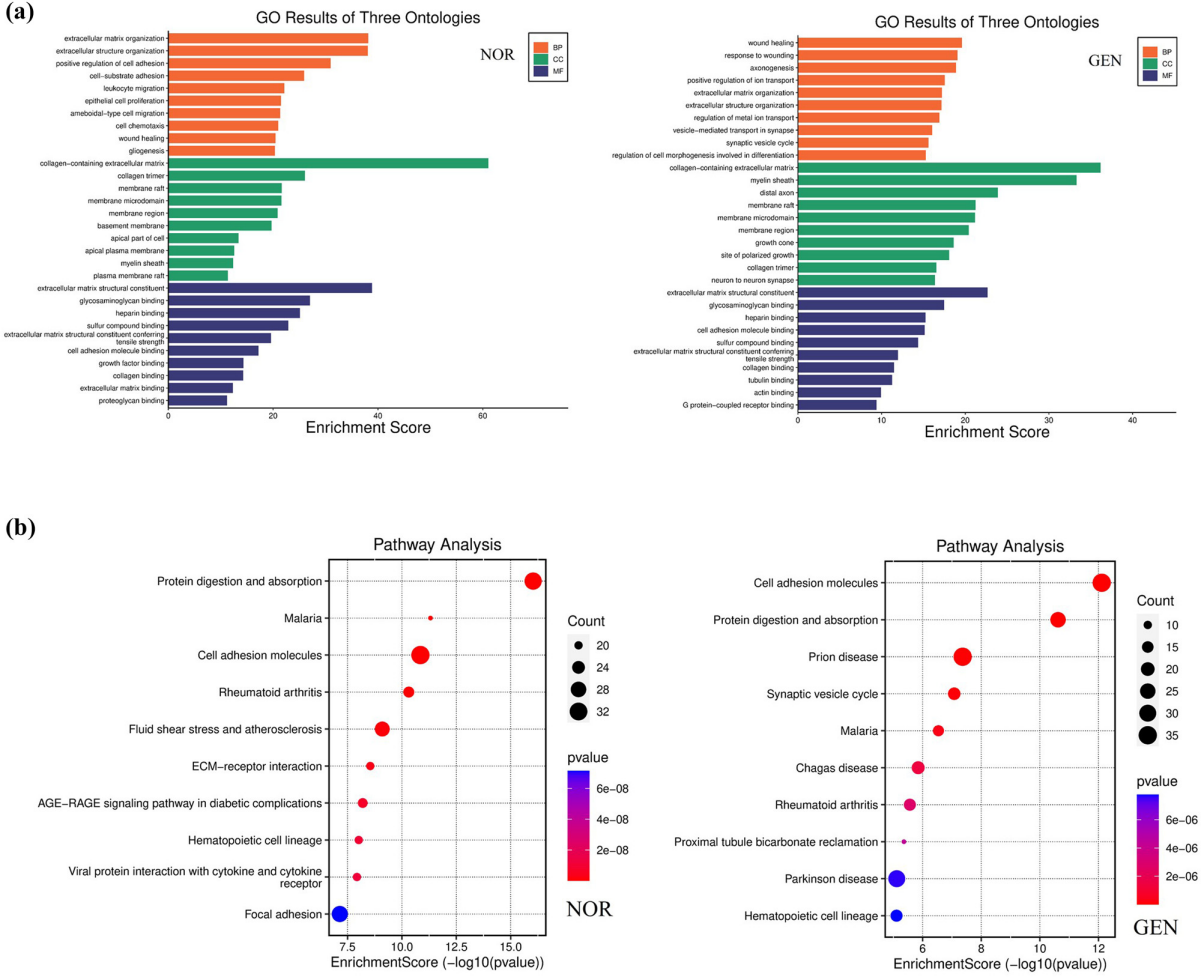
GO and KEGG enrichment analysis of DEGs. (a) GO enrichment analysis of DEGs between GEN and NOR groups. (b) KEGG pathway enrichment analysis of the same DEG set.

KEGG analysis demonstrated that GEN group DEGs were significantly enriched in the p53 signaling pathway, TNF signaling pathway, and MAPK signaling pathway, all closely related to hair cell damage and inflammatory response (*P* < 0.05, Figure 10, Supplementary 2).

#### 3.2.7 Distribution characteristics of nuclear genes

Following batch effect correction using Harmony and cell clustering annotation, 31 cell clusters were identified. DEGs for each cell cluster were selected and visualized with Dotplot to preliminarily identify highly specific marker genes in each cluster and Prestin (Slc26a5) and Myo7a acted as canonical hair cell markers. The results are as follows (Table 2):

**Table 2:** Top ten DEGs in each cell cluster following integration of single-cell data from all three groups

Gene	*p*_val	avg_log2FC	pct.1	pct.2	*p*_val_adj	Cluster
Il1b	0	2.296307	0.874	0.306	0	NEUT1
Ccl6	0	2.230189	0.943	0.357	0	NEUT1
Cxcl2	0	2.137592	0.976	0.551	0	NEUT1
Ccrl2	0	2.130405	0.92	0.516	0	NEUT1
Clec4d	0	2.074705	0.994	0.505	0	NEUT1
Hcar2	0	2.073142	0.937	0.465	0	NEUT1
Nlrp3	0	1.974674	0.946	0.465	0	NEUT1
Acod1	0	1.941379	0.945	0.532	0	NEUT1
Il1r2	0	1.901822	0.957	0.515	0	NEUT1
Csf3r	0	1.899401	0.977	0.583	0	NEUT1
Retnlg	0	2.079243	0.999	0.711	0	NEUT2
Mmp8	0	1.922687	0.995	0.514	0	NEUT2
Fpr1	0	1.508132	0.907	0.39	0	NEUT2
S100a6	0	1.455033	0.979	0.676	0	NEUT2
Il1f9	0	1.407325	0.929	0.438	0	NEUT2
Mmp9	0	1.353328	0.996	0.519	0	NEUT2
Prr13	0	1.297979	0.989	0.764	0	NEUT2
Adam8	0	1.297021	0.99	0.578	0	NEUT2
Thbs1	0	1.231989	0.738	0.42	0	NEUT2
R3hdm4	0	1.21724	0.982	0.774	0	NEUT2
Ltf	0	2.369141	0.986	0.443	0	NEUT3
Ngp	0	2.02572	0.999	0.725	0	NEUT3
Ifitm6	0	1.926356	0.995	0.516	0	NEUT3
Lcn2	0	1.822955	1	0.695	0	NEUT3
Anxa1	0	1.805717	1	0.706	0	NEUT3
Camp	0	1.759623	0.986	0.395	0	NEUT3
Wfdc21	0	1.666808	0.999	0.559	0	NEUT3
Plbd1	0	1.590771	0.972	0.421	0	NEUT3
Cybb	0	1.582672	0.992	0.617	0	NEUT3
Ly6g	0	1.580378	0.977	0.425	0	NEUT3
Rsad2	0	2.767022	0.575	0.172	0	NEUT4
Il1b	0	2.566372	0.938	0.365	0	NEUT4
Ccrl2	0	2.562171	0.961	0.558	0	NEUT4
Ptgs2	0	2.456352	0.639	0.182	0	NEUT4
Ccl3	0	2.412587	0.831	0.434	0	NEUT4
Cxcl2	0	2.222273	0.972	0.598	0	NEUT4
Cd274	0	2.147376	0.616	0.185	0	NEUT4
Csf1	1.74 × 10^−249^	2.254707	0.577	0.22	4.33 × 10^−245^	NEUT4
Ccl4	4.85 × 10^−214^	2.276061	0.511	0.185	1.21 × 10^−209^	NEUT4
Gbp2	1.53 × 10^−100^	2.634624	0.419	0.218	3.81 × 10^−96^	NEUT4
Igkc	0	4.413135	0.889	0.174	0	B1
Ighm	0	4.053927	0.982	0.214	0	B1
Sox4	0	3.720767	0.842	0.127	0	B1
Cd79a	0	3.564249	0.981	0.068	0	B1
Fcrla	0	3.533266	0.814	0.047	0	B1
Ebf1	0	3.497047	0.935	0.093	0	B1
Vpreb3	0	3.475994	0.932	0.05	0	B1
Spib	0	3.323718	0.854	0.064	0	B1
Cecr2	0	3.288302	0.894	0.063	0	B1
Fam129c	0	3.243563	0.843	0.057	0	B1
Chil3	0	2.477715	0.989	0.446	0	NEUT5
Camp	0	2.461097	0.988	0.422	0	NEUT5
Ltf	0	2.376249	0.985	0.467	0	NEUT5
Ngp	0	2.301347	0.994	0.737	0	NEUT5
Zmpste24	0	1.966458	0.813	0.282	0	NEUT5
Lcn2	0	1.958909	0.997	0.709	0	NEUT5
Ifitm6	0	1.90725	0.983	0.538	0	NEUT5
Cybb	0	1.869416	0.991	0.634	0	NEUT5
Anxa1	0	1.690295	0.997	0.719	0	NEUT5
Orm1	0	1.688015	0.749	0.099	0	NEUT5
Chil3	0	3.356882	0.998	0.45	0	NEUT6
Camp	0	2.526071	0.997	0.426	0	NEUT6
Fcnb	0	2.270154	0.832	0.113	0	NEUT6
Hmgn2	0	2.210258	0.993	0.645	0	NEUT6
Ngp	0	2.075144	0.998	0.739	0	NEUT6
Top2a	0	2.022558	0.939	0.187	0	NEUT6
Ube2c	0	2.012327	0.76	0.136	0	NEUT6
Smc4	0	1.900779	0.991	0.41	0	NEUT6
Mki67	0	1.859998	0.975	0.2	0	NEUT6
Hmgb2	0	1.831134	1	0.906	0	NEUT6
F13a1	0	3.848398	0.965	0.052	0	NEUT7
Fn1	0	3.575091	0.932	0.06	0	NEUT7
Ms4a6c	0	3.2431	0.953	0.052	0	NEUT7
Ctss	0	3.069778	0.998	0.181	0	NEUT7
Ccr2	0	3.014537	0.975	0.053	0	NEUT7
Ctsc	0	2.95775	0.991	0.237	0	NEUT7
Ccl9	0	2.94072	0.955	0.066	0	NEUT7
Ahnak	0	2.632812	0.985	0.22	0	NEUT7
Psap	0	2.597219	1	0.924	0	NEUT7
Klf4	0	2.522328	0.929	0.238	0	NEUT7
Ccl5	0	5.335997	0.645	0.022	0	T
Gzma	0	3.902751	0.448	0.004	0	T
Il2rb	0	3.702139	0.833	0.004	0	T
Trac	0	3.575134	0.606	0.005	0	T
Xcl1	0	3.492293	0.516	0.003	0	T
Trbc2	0	3.233065	0.703	0.006	0	T
AW112010	0	3.049239	0.743	0.123	0	T
Nkg7	0	2.791016	0.691	0.079	0	T
Lck	0	2.761573	0.89	0.028	0	T
Rgs1	0	2.74656	0.708	0.061	0	T
Top2a	0	3.014256	0.939	0.204	0	B2
Ezh2	0	2.872195	0.995	0.3	0	B2
Vpreb3	0	2.830962	0.992	0.076	0	B2
Ptma	0	2.761883	1	0.585	0	B2
Hmgb1	0	2.664208	1	0.6	0	B2
Tubb5	0	2.630771	0.992	0.358	0	B2
Stmn1	0	2.600264	0.983	0.234	0	B2
Pclaf	0	2.593159	0.92	0.163	0	B2
Ighm	0	2.564379	0.993	0.237	0	B2
H2afv	0	2.434125	0.983	0.325	0	B2
Hba-a1	0	6.761932	0.96	0.166	0	ERYT1
Hbb-bs	0	6.581102	0.947	0.084	0	ERYT1
Hbb-bt	0	6.126738	0.918	0.037	0	ERYT1
Car2	0	5.755228	0.991	0.1	0	ERYT1
Slc4a1	0	5.243651	0.879	0.013	0	ERYT1
Cpox	0	4.634532	0.935	0.147	0	ERYT1
Gypa	0	4.32368	0.901	0.005	0	ERYT1
Prdx2	0	4.211731	0.972	0.232	0	ERYT1
Aqp1	0	3.916893	0.911	0.021	0	ERYT1
Mki67	0	3.868076	0.975	0.22	0	ERYT1
Ly6c1	0	5.497781	0.967	0.067	0	EC
Flt1	0	5.244363	0.98	0.016	0	EC
Spock2	0	5.181551	0.792	0.031	0	EC
Ly6a	0	4.560383	0.967	0.068	0	EC
Itm2a	0	4.519889	0.767	0.052	0	EC
Ptprb	0	4.503494	0.971	0.004	0	EC
Pltp	0	4.399358	0.9	0.042	0	EC
Cldn5	0	4.363167	0.865	0.002	0	EC
Igfbp7	0	4.323402	0.958	0.031	0	EC
Adgrf5	0	4.276998	0.984	0.005	0	EC
Siglech	0	3.664939	0.609	0.006	0	DC1
Ccr9	0	3.04166	0.405	0.007	0	DC1
Lgals1	0	2.909442	0.982	0.218	0	DC1
Cd7	0	2.5661	0.657	0.022	0	DC1
Irf8	0	2.498057	0.863	0.153	0	DC1
Cox6a2	0	2.293865	0.469	0.009	0	DC1
Cd74	6.72 × 10^−265^	2.685859	0.879	0.219	1.67 × 10^−260^	DC1
Tcf4	3.61 × 10^−256^	2.57813	0.86	0.228	9.00 × 10^−252^	DC1
H2-Aa	4.45 × 10^−184^	2.793063	0.515	0.097	1.11 × 10^−179^	DC1
H2-Ab1	1.37 × 10^−164^	2. 561331	0.481	0.093	3.41 × 10^−160^	DC1
Cd74	0	4.631161	0.974	0.219	0	B3
H2-Aa	0	4.368422	0.774	0.093	0	B3
Igkc	0	4.182451	0.955	0.202	0	B3
H2-Ab1	0	4.098241	0.826	0.087	0	B3
Ms4a1	0	3.857937	0.866	0.009	0	B3
Cd79a	0	3.618734	0.992	0.105	0	B3
Cd83	0	3.183551	0.795	0.104	0	B3
Iglc2	0	3.138845	0.713	0.013	0	B3
Ighm	1.28 × 10^−297^	3.286376	0.966	0.246	3.19 × 10^−293^	B3
H2-Eb1	1.83 × 10^−297^	3.199098	0.574	0.072	4.56 × 10^−293^	B3
Ctla2a	0	3.431689	0.732	0.043	0	Mast
Gata2	0	2.792429	0.668	0.037	0	Mast
Cpa3	0	2.784117	0.343	0.002	0	Mast
Rgs1	0	2.456825	0.67	0.074	0	Mast
Cdk6	6.59 × 10^−303^	2.111122	0.824	0.158	1.64 × 10^−298^	Mast
Ifitm1	2.69 × 10^−200^	2.154224	0.803	0.195	6.70 × 10^−196^	Mast
Rpl32	8.27 × 10^−199^	2.063971	0.989	0.545	2.06 × 10^−194^	Mast
Rps4x	1.23 × 10^−183^	2.053586	0.995	0.65	3.06 × 10^−179^	Mast
H2afy	1.44 × 10^−113^	2.090347	0.943	0.615	3.60 × 10^−109^	Mast
Ccl4	1.55 × 10^−47^	2.254853	0.511	0.199	3.85 × 10^−43^	Mast
Sod3	0	5.568798	0.77	0.025	0	Epi
Krt18	0	4.645166	0.943	0.025	0	Epi
Epcam	0	4.588636	0.986	0.038	0	Epi
Aldh1a1	0	4.416025	0.842	0.032	0	Epi
Hspb1	0	4.318796	0.92	0.074	0	Epi
Krt8	0	4.196297	0.876	0.019	0	Epi
Cldn4	0	4.112089	0.819	0.009	0	Epi
Tmem176b	0	4.064797	0.94	0.119	0	Epi
Timp3	0	3.824602	0.871	0.069	0	Epi
Tmem176a	0	3.822391	0.902	0.095	0	Epi
Fcnb	0	4.308985	0.979	0.136	0	NEUT8
Elane	0	4.034507	0.991	0.128	0	NEUT8
Prtn3	0	4.015441	0.997	0.131	0	NEUT8
Mpo	0	3.745653	0.967	0.103	0	NEUT8
Ms4a3	0	2.589234	0.985	0.075	0	NEUT8
Ctsg	0	2.297662	0.86	0.068	0	NEUT8
Igfbp4	0	2.199301	0.985	0.185	0	NEUT8
Gstm1	1.31 × 10^−272^	2.26708	0.976	0.233	3.26 × 10^−268^	NEUT8
Lta4h	2.20 × 10^−219^	2.179699	0.985	0.397	5.47 × 10^−215^	NEUT8
Serpinb1a	1.29 × 10^−204^	2.343175	0.967	0.384	3.20 × 10^−200^	NEUT8
Mpo	0	5.599578	1	0.103	0	NEUT9
Elane	0	5.508864	0.991	0.128	0	NEUT9
Prtn3	0	5.064998	0.997	0.131	0	NEUT9
Ctsg	0	4.421696	0.997	0.066	0	NEUT9
Gstm1	0	3.344881	0.997	0.233	0	NEUT9
Ms4a3	0	3.313892	0.997	0.075	0	NEUT9
Nkg7	0	2.83007	0.994	0.087	0	NEUT9
Fcnb	6.02 × 10^−254^	2.89561	0.784	0.139	1.50 × 10^−249^	NEUT9
Plac8	6.13 × 10^−209^	2.799115	1	0.487	1.53 × 10^−204^	NEUT9
Calr	3.32 × 10^−202^	2.740057	1	0.607	8.27 × 10^−198^	NEUT9
Col1a2	0	7.019246	0.874	0.05	0	Fibr
Sparc	0	5.429979	0.976	0.086	0	Fibr
Apod	0	5.414049	0.417	0.017	0	Fibr
Dcn	0	5.353722	0.76	0.005	0	Fibr
Igfbp5	0	5.224145	0.728	0.023	0	Fibr
Ibsp	0	4.879163	0.331	0.003	0	Fibr
Cxcl12	0	4.216762	0.492	0.024	0	Fibr
Col3a1	0	3.903568	0.583	0.005	0	Fibr
Col1a1	2.31 × 10^−284^	7.387203	0.689	0.084	5.76 × 10^−280^	Fibr
Ptgds	2.91 × 10^−37^	4.747365	0.276	0.074	7.25 × 10^−33^	Fibr
Mpo	0	3.564739	0.989	0.109	0	Mono1
F13a1	0	2.742456	0.995	0.089	0	Mono1
Ctsg	0	2.494044	1	0.072	0	Mono1
Ms4a6c	0	2.284719	0.995	0.088	0	Mono1
Prtn3	8.53 × 10^−282^	3.03184	1	0.137	2.12 × 10^−277^	Mono1
Cxcl10	5.68 × 10^−194^	2.58553	0.934	0.16	1.41 × 10^−189^	Mono1
Lgals1	1.57 × 10^−188^	2.709256	1	0.228	3.91 × 10^−184^	Mono1
Plac8	1.16 × 10^−115^	2.598258	1	0.49	2.88 × 10^−111^	Mono1
H2afy	1.35 × 10^−113^	2.518226	1	0.617	3.37 × 10^−109^	Mono1
Npm1	4.53 × 10^−110^	2.273215	1	0.511	1.13 × 10^−105^	Mono1
Ahnak	6.02 × 10^−77^	2.320335	0.797	0.253	1.50 × 10^−72^	Mono2
Gm26917	2.23 × 10^−66^	4.469736	0.571	0.16	5.56 × 10^−62^	Mono2
mt-Nd1	1.16 × 10^−56^	2.309783	0.983	0.747	2.88 × 10^−52^	Mono2
mt-Atp6	4.78 × 10^−53^	2.272857	1	0.907	1.19 × 10^−48^	Mono2
mt-Co3	1.37 × 10^−52^	2.383037	0.989	0.867	3.41 × 10^−48^	Mono2
Gm47283	3.48 × 10^−49^	2.464701	0.78	0.377	8.66 × 10^−45^	Mono2
Hspa1a	4.86 × 10^−42^	2.564324	0.52	0.169	1.21 × 10^−37^	Mono2
Cd74	5.99 × 10^−37^	2.841195	0.576	0.23	1.49 × 10^−32^	Mono2
H2-Aa	1.46 × 10^−26^	2.417142	0.339	0.104	3.64 × 10^−22^	Mono2
H2-Ab1	8.68 × 10^−22^	3.144168	0.299	0.099	2.16 × 10^−17^	Mono2
Nnat	0	6.004872	0.738	0.015	0	OHC
Dlk1	0	5.616719	0.64	0.019	0	OHC
Chgb	0	5.293739	0.849	0.004	0	OHC
Meg3	0	4.986895	0.89	0.042	0	OHC
Scg2	0	4.377325	0.837	0.003	0	OHC
Pcsk1n	0	4.219726	0.919	0.008	0	OHC
Scg5	0	3.727801	0.93	0.016	0	OHC
Mt2	1.66 × 10^−87^	4.415268	0.814	0.272	4.14 × 10^−83^	OHC
Gh	3.68 × 10^−86^	8.454414	0.692	0.166	9.16 × 10^−82^	OHC
Prl	1.16 × 10^−85^	8.370354	0.424	0.063	2.90 × 10^−81^	OHC
Tagln	0	5.881273	0.993	0.012	0	SMC
Myh11	0	5.459393	1	0.003	0	SMC
Sparcl1	0	5.442591	1	0.048	0	SMC
Cald1	0	5.404352	1	0.044	0	SMC
Mylk	0	5.299315	1	0.032	0	SMC
Myl9	0	5.18155	1	0.01	0	SMC
Rgs5	0	5.142747	0.646	0.008	0	SMC
Igfbp7	0	4.757058	1	0.045	0	SMC
Tpm1	3.01 × 10^−236^	5.111376	1	0.153	7.50 × 10^−232^	SMC
Acta2	2.69 × 10^−185^	5.673155	0.993	0.206	6.71 × 10^−181^	SMC
C1qc	0	5.054586	0.97	0.003	0	Mac
C1qb	0	4.399197	0.985	0.002	0	Mac
C1qa	0	4.208365	0.978	0.001	0	Mac
Apoe	1.76 × 10^−201^	5.181583	0.888	0.113	4.39 × 10^−197^	Mac
Ctss	3.54 × 10^−160^	4.210803	1	0.216	8.82 × 10^−156^	Mac
Hexb	9.40 × 10^−114^	4.133809	0.94	0.275	2.34 × 10^−109^	Mac
Hspa1a	1.60 × 10^−98^	4.162597	0.776	0.168	3.98 × 10^−94^	Mac
Cd74	3.72 × 10^−94^	4.039485	0.873	0.229	9.26 × 10^−90^	Mac
Hspa1b	1.65 × 10^−93^	4.112077	0.799	0.183	4.10 × 10^−89^	Mac
Ccl4	3.32 × 10^−89^	4.251913	0.799	0.2	8.26 × 10^−85^	Mac
Mpz	0	7.79169	0.878	0.01	0	Schw
Pmp22	0	5.492076	0.962	0.039	0	Schw
Plp1	0	4.780818	0.992	0.022	0	Schw
Cryab	0	3.844024	0.939	0.045	0	Schw
Mal	0	3.824259	0.931	0.039	0	Schw
Igfbp6	0	3.815037	0.45	0.011	0	Schw
Kcna1	0	3.745437	0.847	0.004	0	Schw
Scd2	8.83 × 10^−194^	4.816866	1	0.168	2.20 × 10^−189^	Schw
Mbp	1.11 × 10^−109^	4.330123	0.954	0.304	2.77 × 10^−105^	Schw
Apoe	2.18 × 10^−24^	4.129665	0.389	0.117	5.42 × 10^−20^	Schw
Dnase1	0	5.292244	0.357	0.003	0	Stria-border
Enpep	0	4.539279	0.986	0.031	0	Stria-border
Lrp2	0	4.342953	0.986	0.007	0	Stria-border
Gas2	0	4.168965	0.957	0.024	0	Stria-border
Dclk1	0	4.027127	0.971	0.029	0	Stria-border
Gpx3	5.84 × 10^−255^	4.562391	0.986	0.058	1.46 × 10^−250^	Stria-border
Atp1b2	2.67 × 10^−229^	6.197312	1	0.068	6.64 × 10^−225^	Stria-border
Ptgds	2.88 × 10^−213^	6.358935	1	0.073	7.16 × 10^−209^	Stria-border
Slc12a2	4.65 × 10^−204^	5.600001	1	0.077	1.16 × 10^−199^	Stria-border
Atp1a1	5.64 × 10^−65^	4.404918	1	0.34	1.41 × 10^−60^	Stria-border
Lgals7	0	4.824722	0.606	0.007	0	Stria-basal
Krt5	0	4.822037	0.697	0.002	0	Stria-basal
Krt14	0	4.793011	0.667	0.002	0	Stria-basal
Cyp2f2	0	4.789308	0.47	0.003	0	Stria-basal
Krt15	0	3.727416	0.621	0.001	0	Stria-basal
Dsp	0	3.701699	0.955	0.03	0	Stria-basal
Abi3bp	1.97 × 10^−292^	3.756986	0.439	0.008	4.90 × 10^−288^	Stria-basal
Krt1	2.09 × 10^−235^	4.324499	0.288	0.004	5.21 × 10^−231^	Stria-basal
Bpifa1	2.58 × 10^−166^	7.675366	0.409	0.013	6.42 × 10^−162^	Stria-basal
Perp	2.48 × 10^−143^	3.96785	0.955	0.095	6.18 × 10^−139^	Stria-basal
Dct	0	7.754018	0.964	0.005	0	Stria-int
Gsta4	0	5.431319	1	0.036	0	Stria-int
Gpnmb	0	4.870221	0.964	0.007	0	Stria-int
Gjb6	0	4.782277	0.964	0.006	0	Stria-int
Dkk3	0	4.764847	0.964	0.023	0	Stria-int
Ptgds	5.15 × 10^−171^	5.846893	1	0.074	1.28 × 10^−166^	Stria-int
Hpse	4.20 × 10^−154^	4.975566	0.964	0.077	1.05 × 10^−149^	Stria-int
Slc12a2	4.50 × 10^−144^	4.876733	0.946	0.078	1.12 × 10^−139^	Stria-int
Atp1b1	9.40 × 10^−77^	6.169884	1	0.189	2.34 × 10^−72^	Stria-int
Atp1a1	1.30 × 10^−51^	4.6746	1	0.34	3.23 × 10^−47^	Stria-int
Alas2	3.80 × 10^−178^	4.639409	0.692	0.028	9.46 × 10^−174^	ERYT2
Hba-a2	8.36 × 10^−177^	5.912645	0.654	0.026	2.08 × 10^−172^	ERYT2
Hbb-bt	2.01 × 10^−150^	6.383028	0.885	0.058	5.01 × 10^−146^	ERYT2
Snca	3.82 × 10^−130^	3.519117	0.577	0.027	9.51 × 10^−126^	ERYT2
Hbb-bs	1.14 × 10^−96^	6.471828	0.923	0.104	2.84 × 10^−92^	ERYT2
Hba-a1	3.76 × 10^−68^	6.565205	0.981	0.184	9.36 × 10^−64^	ERYT2
Bpgm	3.23 × 10^−62^	3.488751	0.538	0.051	8.05 × 10^−58^	ERYT2
Fech	4.01 × 10^−31^	4.045983	0.615	0.134	1.00 × 10^−26^	ERYT2
Tent5c	2.97 × 10^−19^	3.63628	0.654	0.236	7.39 × 10^−15^	ERYT2
Mkrn1	6.39 × 10^−14^	3.270593	0.75	0.463	1.59 × 10^−09^	ERYT2
Plp1	0	8.631235	1	0.026	0	Glial cell
Aplp1	0	6.191495	1	0.025	0	Glial cell
Tubb4a	0	6.185396	1	0.025	0	Glial cell
Mag	0	5.740784	1	0.014	0	Glial cell
Cldn11	0	5.729523	1	0.01	0	Glial cell
Mal	4.01 × 10^−230^	5.340918	1	0.043	9.99 × 10^−226^	Glial cell
Syt11	1.05 × 10^−212^	5.388431	1	0.047	2.62 × 10^−208^	Glial cell
Enpp2	1.86 × 10^−211^	5.084028	1	0.047	4.62 × 10^−207^	Glial cell
Scd2	8.20 × 10^−69^	5.230188	1	0.171	2.04 × 10^−64^	Glial cell
Mbp	1.58 × 10^−45^	5.137117	1	0.306	3.94 × 10^−41^	Glial cell
Prlr	0	4.387577	0.955	0.008	0	IHC
Igfbp2	0	4.374309	1	0.012	0	IHC
1500015O10Rik	0	4.269537	1	0.007	0	IHC
Kl	0	3.985654	1	0.004	0	IHC
2900040C04Rik	0	3.664745	1	0.001	0	IHC
Car12	6.04 × 10^−308^	3.42191	1	0.015	1.50 × 10^−303^	IHC
Clu	1.19 × 10^−106^	4.345548	1	0.046	2.96 × 10^−102^	IHC
Enpp2	5.49 × 10^−105^	8.203794	1	0.048	1.37 × 10^−100^	IHC
Slc22a17	3.77 × 10^−99^	4.114721	1	0.05	9.40 × 10^−95^	IHC
Ttr	4.09 × 10^−96^	9.836147	1	0.053	1.02 × 10^−91^	IHC
Myo7a	5.91 × 10^−57^	0.821842364	0.818	0.053	1.47 × 10^−52^	IHC
Slc26a5	1.02 × 10^−24^	1.895397361	0.955	0.196	2.53 × 10^−20^	IHC

B cells: “Fcrla”. “Vpreb3”. “Cecr2”. “Fam129c”. “Cd79a”. Dendritic cells: “Siglech”. “Ccr9”. “Cd7”. “Cox6a2”. “Ly86”. Endothelial cells: “Ly6c1”. “Flt1”. “Ptprb”. “Cldn5”. “Adgrf5”. Epithelial cells: “Sod3”. “Krt18”. “Epcam”. “Aldh1a1”. “Hspb1”. Red blood cells: “Hba-a1”. “Hbb-bs”. “Slc4a1”. “Gypa”. “Aqp1”. Fibroblasts: “Col1a1”. “Col1a2”. “Apod”. “Dcn”. “Igfbp5”. Glial Cells: “Mag”. “Cldn11”. “Mobp”. “Ermn”. “Efnb3”. Inner hair cells: “Ttr”. “Enpp2”. “Prlr”. “Igfbp2”. “Clu”. Macrophages: “C1qc”. “C1qb”. “C1qa”. “Hspa1a”. “Hspa1b”. Mast cells: “Cd34”. “Slc18a2”. “Rps20”. “Rpl5”. “Eef1a1”. Monocytes: “Ass1”. “Pld4”. “Itga5”. “Ccdc88a”. Neutrophils: “Rsad2”. “Gbp2”. “Il1b”. “Ccrl2”. “Chil3”. Outer hair cells: “Nnat”. “Dlk1”. “Chgb”. “Meg3”. “Scg2”. Schwann cells: “Mpz”. “Art3”. “Slc6a15”. “Ncmap”. Smooth muscle cells: “Tagln”. “Myh11”. “Rgs 5”. “Mustn1”. “Notch3”. Strial basal cells: “Bpifa1”. “Lgals7”. “Krt5”. “Krt14”. “Cyp2f2”. Strial marginal cells: “Ptgds”. “Atp1b2”. “Slc12a2”. “Dnase1”. “Gpx3”. Strial intermediate cells: “Dct”. “Hpse”. “Gpnmb”. “Dkk3”. “Slc45a2”. T Cells: “Ccl5”. “Gzma”. “Il2rb”. “Trac”. “Xcl1”.

### 3.3 QC and sample filtering

To ensure high data quality and minimize contamination from blood-derived cells, we implemented a multi-step QC pipeline based on both global metrics and cell-type markers. First, we computed the following per-cell metrics: number of detected genes (nFeature_RNA), total UMI counts (nCount_RNA), mitochondrial transcript fraction (percent.mt), ribosomal transcript fraction (percent_ribo), and hemoglobin transcript fraction (percent_Hb). Cells satisfying 500 < nFeature_RNA < 6 000, nCount_RNA < 25,000, percent.mt < 20%, percent_ribo > 1%, and percent_Hb < 1% were retained for downstream analysis.

Next, we quantified expression of canonical neutrophil markers (e.g., Ly6g, Mpo) and confirmed that contaminating neutrophil-like cells accounted for <1% of all barcodes post-filtering.

Finally, batch effects arising from three experimental groups (NOR, GEN, DEX) were corrected using the Harmony algorithm, ensuring that downstream clustering reflects true biological variance rather than technical confounders.

## 4 Discussion

The study employed scRNA-seq technology to systematically analyze the impact of GEN intraperitoneal injection on the inner ear cells of mice, revealing characteristic changes across different cell types involved in ototoxic damage. The inner ear is a critical organ for auditory and vestibular functions, involving the coordination of various cell types such as hair cells, supporting cells, and strial vascular cells to maintain auditory function stability [27,28]. However, aminoglycoside antibiotics, such as GEN, can cause irreversible cochlear hair cell damage, leading to permanent hearing loss and vestibular dysfunction, whose underlying molecular mechanisms remain to be explored [29,30]. Traditional RNA sequencing methods, due to their reliance on tissue homogenization, cannot resolve the transcriptional features of specific cell types [4], whereas scRNA-seq technology overcomes this limitation, allowing researchers to reveal the gene expression patterns and drug responses of cochlear cells at single-cell resolution [31,32].

The study discovered, through ABR testing, that the auditory threshold in the GEN group was significantly higher than in the NOR group, while DEX group intervention partially improved the threshold, indicating that GEN significantly damages auditory function, and glucocorticoid drugs might have a protective effect on the cochlea. Further single-cell sequencing analysis revealed significant changes in the composition of cochlear hair cells, supporting cells, and immune cells in mice from the GEN group. Compared to the NOR group, the proportion of hair cells significantly decreased, whereas the proportion of T cells, B cells, macrophages, and Schwann cells significantly increased, suggesting that hair cell damage might be accompanied by activation of the inflammatory response. Furthermore, DEG analysis showed that genes specific to hair cells such as Nnat and Dlk1 were significantly downregulated in the GEN group, while genes related to the complement system, such as C1qa, C1qb, and C1qc, were notably upregulated, indicating a possible enhancement of local immune response accompanying hair cell damage.

This study applied scRNA-seq using the 10x Genomics platform with Seurat v4 for data processing, focusing on rigorous QC, UMAP clustering, and pathway analysis using clusterProfiler. This approach differs from previous study [33], which integrated snRNA-seq and scRNA-seq with Cell Ranger and performed trajectory analysis (Monocle, RNA velocity). Yan et al. used the Fluidigm C1 system for precise single-cell capture and performed pathway analysis with Metascape and transcription factor analysis via Cytoscape [34]. Compared to these studies, our method avoids complex integration and trajectory analysis but ensures robust cell-type classification and DEG validation using complementary techniques. This streamlined approach is suitable for capturing cell-type-specific responses in our acute injury model. Although minor blood cell contamination can occur during cochlear dissociation, our rigorous QC thresholds and marker-based exclusion reduced neutrophil-derived barcodes to negligible levels (<1%). We therefore conclude that residual neutrophil content does not compromise the integrity of our single-cell transcriptomic analyses.

Studies have shown that aminoglycoside antibiotics can bind to mitochondrial rRNA, inhibiting electron transport chain (ETC) complex activity, leading to increased reactive oxygen species levels, thereby causing mitochondrial oxidative damage [35]. The study found that genes related to OXPHOS, such as ND1, ND2, and CYTB, were significantly downregulated in the cochlea of GEN group mice, suggesting that mitochondrial dysfunction could be a crucial factor in hair cell death. Additionally, KEGG pathway analysis revealed significant activation of the p53 signaling pathway, TNF signaling pathway, and MAPK signaling pathway in the cochlea of GEN group mice. The p53 signaling pathway plays a vital role in apoptosis, and its upregulation can induce an imbalanced Bax/Bcl-2 ratio, activating the mitochondrial-dependent apoptotic pathway, ultimately leading to hair cell death [36,37]. Concurrently, activation of the TNF signaling pathway may exacerbate inflammatory responses, further deteriorating the cochlear microenvironment, accelerating hair cell loss [38,39].

The study’s scRNA-seq results also revealed GEN-induced immune-inflammatory responses. The proportions of macrophages, B cells, and T cells were significantly increased in the GEN group mice, with notable upregulation of C1q complement system genes (C1qa, C1qb, C1qc), suggesting that GEN might enhance local immune responses by activating the complement system and releasing inflammatory factors [40,41]. Previous studies have indicated that excessive activation of the complement system may disrupt the cochlear microenvironment, exacerbating hair cell apoptosis and worsening hearing loss [42].

Our findings align with and expand upon previous scRNA-seq studies investigating ototoxicity, providing further insight into the cellular and molecular mechanisms involved. For example, a study on the SV of the cochlea identified downregulation of mitochondrial ETC pathways as a common feature across various forms of sensorineural hearing loss, implicating mitochondrial dysfunction as a central contributor to SV impairment [43]. Another study using single-cell transcriptomics revealed the complex cellular landscape of the middle ear, highlighting the roles of innate immune responses, particularly among resident monocytes/macrophages, in mediating inflammation and tissue repair [44]. In the context of drug-induced ototoxicity, scRNA-seq analysis of cisplatin-treated SV identified transcriptional alterations affecting genes critical to EP generation and suggested potential therapeutic targets, including Alcam, Atp1b2, and Spp1, which could mitigate cisplatin-induced damage [45]. These findings collectively emphasize that while mitochondrial dysfunction and immune regulation are central to ototoxicity, this study provides a more nuanced view by directly analyzing the transcriptional responses of cochlear cells exposed to different treatments.

Moreover, the study found that after DEX intervention, the expression levels of some inflammation-related genes decreased, with the proportion of hair cells partially restored, indicating that glucocorticoids might have a protective role against GEN ototoxicity by suppressing inflammatory responses. An *in vitro* study using 3-day-old rat organ of Corti explants exposed to TNFα revealed that DXM effectively mitigated TNFα-induced hair cell loss. Specifically, DXM treatment counteracted TNFα-induced upregulation of the pro-apoptotic gene Bax and restored the expression of anti-apoptotic genes Bcl-2 and Bcl-xl [46]. These protective effects were also observed in DXM-eluting biopolymers (SIBS), which prevented hair cell death and maintained the anti- and pro-apoptotic gene expression profile similar to that of DXM-treated explants [47]. Furthermore, recent studies using an inner ear simulating system demonstrated that PSD-NPs loaded with DXM, particularly under a magnetic field, provided enhanced otoprotection compared to DXM alone, significantly reducing hearing loss through anti-apoptotic pathways [48]. However, the protective effect of DEX remains limited, with its precise mechanisms requiring further investigation.

Integrating AlphaFold with our scRNA-seq data can enhance understanding of GEN-induced ototoxicity. AlphaFold’s AI-driven structure prediction can reveal 3D configurations of key proteins, clarify protein–protein interactions, and identify GEN’s binding sites. It also helps explore how DEX modulates protective proteins, uncovering its anti-inflammatory and anti-apoptotic effects. By bridging transcriptomics and protein function, AlphaFold aids in discovering novel therapeutic targets and refining drug designs. This approach offers a high-precision framework for mitigating aminoglycoside-induced hearing loss [49].

Despite the insights provided by this study into the key cell types and molecular mechanisms underlying GEN ototoxicity, certain limitations remain. First, while single-cell sequencing offers high-resolution cellular transcriptome information, experimental procedures such as tissue dissociation and cell capture may result in the loss or bias of some cellular information. For instance, the inability to detect Prestin protein mRNA in this study may be related to its limited molecular structure and quantity. Moreover, this study primarily examines the short-term ototoxic effects following intraperitoneal injection of GEN; future research is required to explore its long-term mechanisms, including variability in ototoxicity across different doses and modes of drug administration. Another limitation of this study is the lack of frequency-specific ABR threshold data (e.g., 8–32 kHz), which would have offered more detailed insights into frequency-dependent hearing loss. Future studies should incorporate full-spectrum ABR evaluations to better characterize cochlear function across the tonotopic axis. In addition, due to pooling of ten cochleae per group into single libraries, differential abundance analyses should be interpreted with caution. Future studies will employ multiple biological replicates to validate these findings. We acknowledged the short-term nature of our exposure model and proposed long-term studies in future work to better reflect chronic clinical scenarios. Finally, although this study identifies numerous DEGs and signaling pathways through bioinformatic analysis, further experimental verification is necessary, such as evaluating the expression levels of key genes using techniques like western blot, qRT-PCR, and immunofluorescence staining, alongside cellular functional assays to explore their specific roles in GEN-induced ototoxicity.

In addition, our single-cell data provide gene-level insights into GEN ototoxicity. Future studies could leverage AI-driven structure prediction tools such as AlphaFold to map DEGs (e.g., complement gene C1q) onto 3D protein structures for drug–target interaction simulations [50]. For instance, GEN may disrupt C1q oligomerization, leading to complement activation, while direct binding to mitochondrial ETC proteins could underlie OXPHOS gene inhibition. Combining computational docking, molecular dynamics, and cryo-EM validation will facilitate structure-based design of otoprotective agents, accelerating translation from mechanistic discovery to clinical intervention [51].

## 5 Conclusion

To conclude, this study systematically elucidates the effects of GEN on different cell types in the mouse inner ear using scRNA-seq technology, revealing that hair cell damage, mitochondrial dysfunction, and immune-inflammatory responses may be pivotal mechanisms in its ototoxic pathway. DEX intervention is shown to partially alleviate hair cell damage, yet its mechanism of action and long-term protective effects warrant further investigation. The findings present potential biomarkers for early detection and targeted therapy of ototoxic damage and provide theoretical support for future ototoxicity prevention and treatment strategies.

## Supplementary Material

Supplementary material

Supplementary Table
