# Supplementary material for: Single-cell RNA sequencing data analysis of the inner ear in gentamicin-treated mice via intraperitoneal injection
Source: Open Med (Wars). 2025 Nov 21;20(1):20251242. doi: 10.1515/med-2025-1242 (PMC12658731; doi:10.1515/med-2025-1242)

Supplementary material

S1 Observation of mouse body weight changes

The body weight of the mice was monitored daily. The average body weight of each group before and after the experiment was recorded, as shown below:

Table S1: The average body weight of the mice in the three groups

Group	Average Body Weight on Day 1 (g)	Average Body Weight on Day 10 (g)
GEN	35.29166667	32.63333333
DEX	33.51666667	31.7
NOR	33.525	33.62727273

S2 Tail suspension landing reflex

S2.1 Method

A vertical background board (50 cm high) was used, with markings at 10 cm intervals. Alternatively, white paper can be affixed to a wall, with lines drawn from the tabletop upwards for reference. Bedding was placed in the cage, and each mouse’s tail was marked with an ink pen for easy identification. The mouse was grasped at the tail's end (about 2 cm from the tip) and quickly lifted to a height of approximately 40–50 cm before being released to fall freely. This procedure was repeated 3–5 times to observe head, neck, trunk, forelimb, and hindlimb extension, as well as muscle tone changes.

S2.2 Evaluation criteria

Normal reflex (0 points): Full extension of the head, neck, trunk, forelimbs, and hindlimbs.

Mildly abnormal reflex (2 points): Partial extension with no relaxation; mild rotation or swaying of the body.

Severely abnormal reflex (4 points): No extension or even contraction of the head, neck, trunk, and limbs; muscle relaxation with significant rotation or tilting of the body.

S3 Aerial righting reflex

Using the same background board as the tail suspension reflex test, a 5 cm thick sponge or extra bedding was placed in the cage to cushion falls. Mice were dropped from a height of 40 cm with their backs facing downward. Observations focused on how the head and trunk adjusted their orientation upon landing and how the limbs contacted the ground.

Table S2: The results of tail lifting and landing reflex analysis in three groups of mice

Group	Outcome	Tail suspension reflex (%)	Abdominal/Trunk extension (%)	Forelimb extension (%)	Hindlimb extension (%)	Neck extension (%)	Muscle tone (%)
GEN	Normal	73.21	62.50	79.46	91.07	66.96	55.36
	Abnormal	26.79	37.50	20.54	8.93	33.04	44.64
DEX	Normal	84.56	69.12	93.38	99.26	77.94	69.12
	Abnormal	15.44	30.88	6.62	0.74	44.12	30.88
NOR	Normal	100.00	94.05	100.00	100.00	100.00	98.81
	Abnormal	0.00	5.95	0.00	0.00	0.00	1.19

Table S3: The results of aerial righting reflex in the three groups of mice

Group	Average righting time (ms)
NOR	125.5
GEN	144.97
DEX	141.36

To enhance the analysis, a Xiaomi smartphone camera was used in slow-motion mode at 240 frames per second (fps) and 720p resolution. The same parameters were maintained throughout the experiment. Tail suspension reflex was recorded from the side. Aerial righting reflex was recorded from the front. Videos were analyzed using Kinovea (www.kinovea.org) with semi-automatic tracking of three points (nose, neck, and tail base) to monitor motion. Timing was recorded from the moment of release to full righting. If the mouse landed on its side or back instead of with all four limbs, the reflex was classified as abnormal.

S3.1 Evaluation criteria

Normal reflex: The head and trunk quickly rotate upright, the neck lifts, and all four limbs extend upon landing.

Abnormal reflex: The head adjusts to the correct position, but the neck and limbs do not fully extend, or the trunk/neck contacts the ground first instead of the limbs.

S4 Rotarod test

S4.1 Method

One day before the formal test, mice underwent training for adaptation (1-2 minutes per session, 2–3 sessions).

A rotarod apparatus was set to an accelerating mode, starting at 4 rotations per minute (rpm) and increasing at 0.5 rpm per second until the mouse fell off. Each group consisted of six mice, and the test was repeated multiple times. Data were analyzed using ANOVA (analysis of variance).

The following parameters were recorded:

Time from rotation start to fall;

Table S4: The results of the rod rotation experiment in three groups of mice

Group	Average falling time (s)
NOR	42.96
DEX	47.20
GEN	27.65

Distance traveled;

Rotation speed at fall.

S5 Histopathological examination of the cochlea (HE Staining)

The cochlear samples were fixed and washed with PBS to remove residual formaldehyde. Samples were then transferred to a 10% EDTA solution for decalcification at 4°C for approximately 72 h. Intermittent shaking or continuous agitation was used to ensure even decalcification.

The paraffin embedding and sectioning process included:

1. Fixation;
2. Dehydration;
3. Clearing;
4. Paraffin infiltration;
5. Embedding;
6. Sectioning;
7. Mounting;
8. Hematoxylin and Eosin (HE) staining.

(Images are included in the supplementary file.)

S6 Scanning electron microscopy (SEM) analysis

Cochlear samples were fixed in 2.5% glutaraldehyde and fully decalcified. They were then dehydrated in a graded ethanol series, dried, and coated with gold. SEM imaging parameters were adjusted, and images were collected and saved.

(Images are included in the supplementary file.)

Supplementary 3

Upregulated genes

Downregulated genes

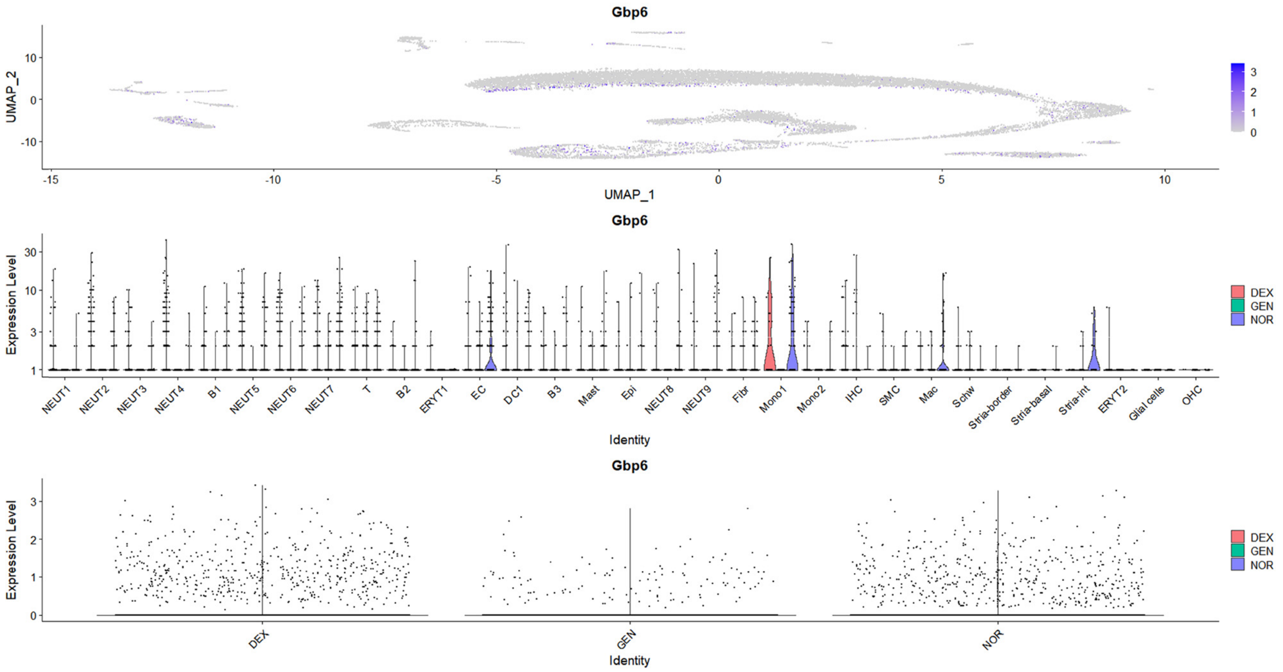

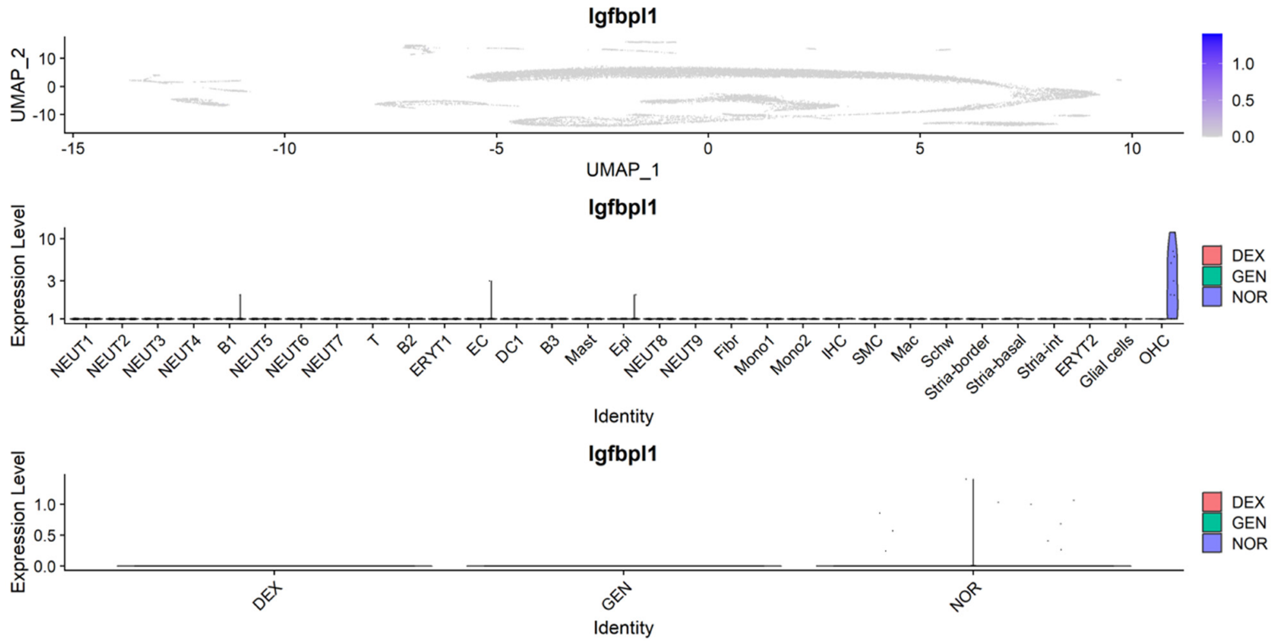

Downregulated genes

Downregulated genes

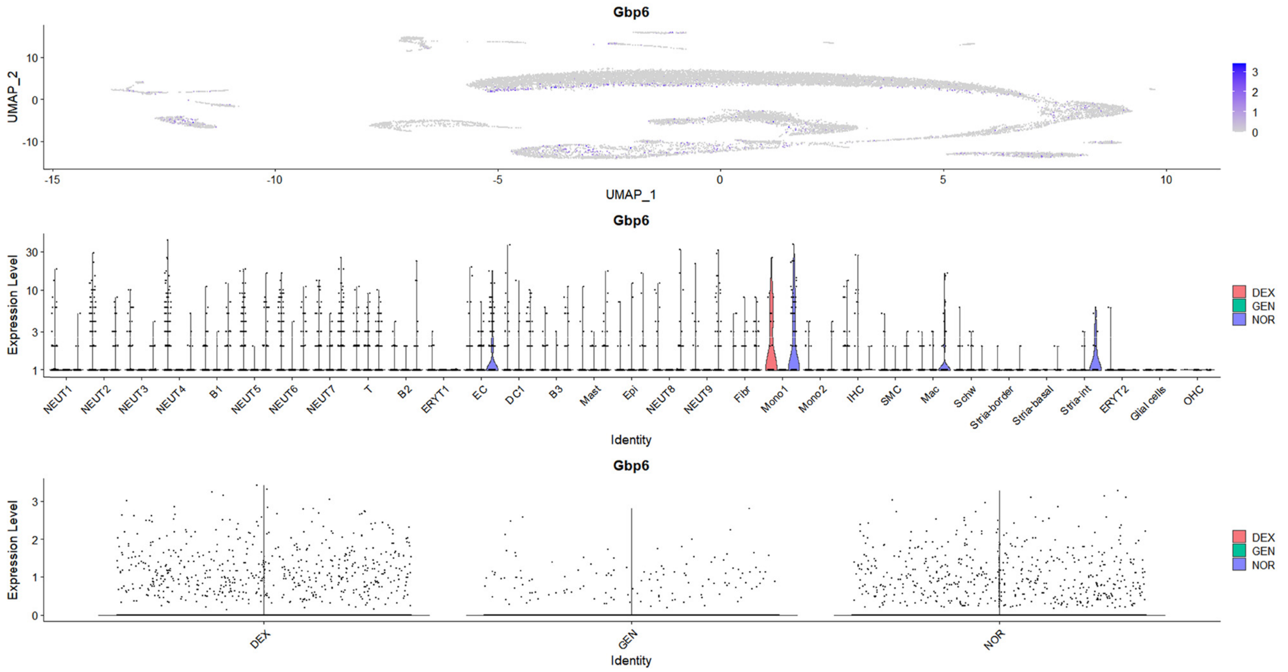

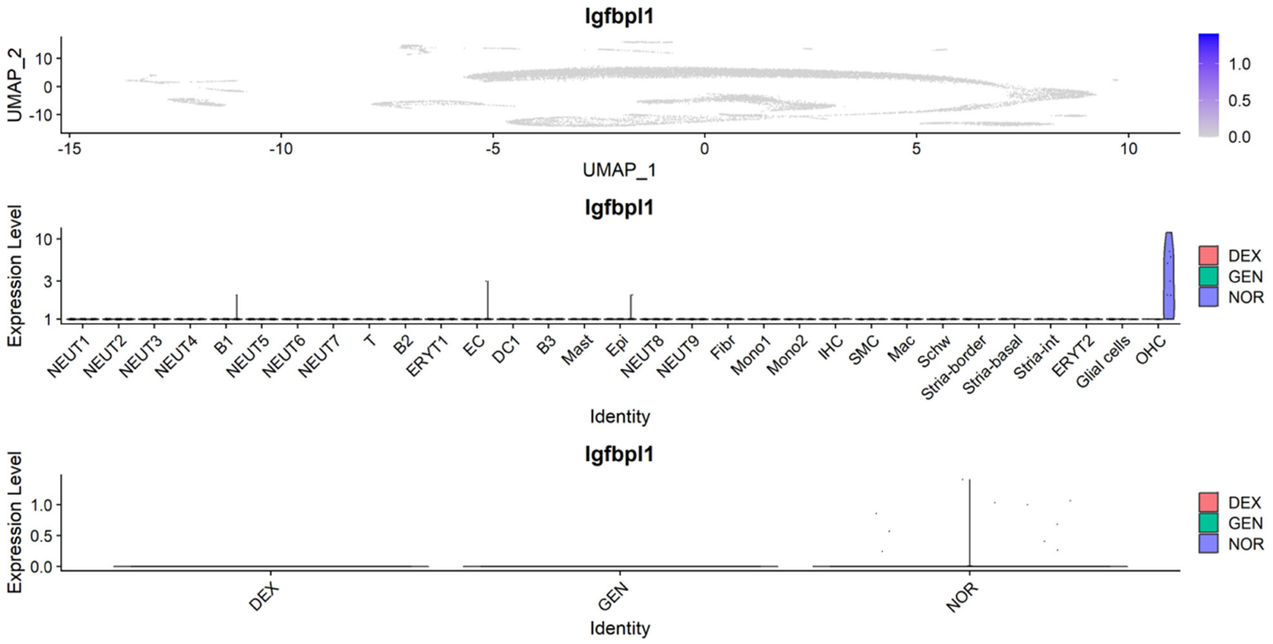

Supplement: Supplementary material [file med-2025-1242-sm.pdf]
